# The energetics of high temperature oxidation and formation mechanisms in binary lanthanide nitrides (*Ln*N)

**DOI:** 10.1038/s42004-026-02236-8

**Published:** 2026-10-07

**Authors:** P. Uhlemann, W. Vance, M. P. Heaney, N. Yaw, C. Schreinemachers, P. Kegler, P. Höhn, X. Guo, G. Modolo, G. L. Murphy

**Affiliations:** 1 Institute of Fusion Energy and Nuclear Waste Management (IFN-2), Forschungszentrum Jülich GmbH, Jülich, Germany; 2 Department of Chemistry, Alexandra Navrotsky Institute for Experimental Thermodynamics, Washington State University, Pullman, WA USA; 3 Max-Planck-Institute for Chemical Physics of Solids, Dresden, Germany

**Keywords:** Thermodynamics, Nuclear waste

## Abstract

Nitride-based materials present exceptional thermal and structural properties for advanced material applications, however accurate determination of their thermodynamic properties is critical to their deployment. Herein, the standard enthalpies of formation, Δ*H*_f_^°^, for all *Ln*N compounds (*Ln* = La, Ce, Pr, Nd, Sm, Eu, Gd, Tb, Dy, Ho, Er, Tm, Lu; excluding Pm) are determined via high temperature oxide melt calorimetry. These measurements are supported by high temperature oxidation studies of La, Ce, Sm, Eu, Er, Yb bearing *Ln*N compounds using X-ray diffraction and thermogravimetric analysis, and are cross-examined against recent high temperature measurements on other *Ln*N compounds^1^. It was determined that Δ*H*_f_^°^ systematically correlates with the ionic radius of *Ln*^3+^ cations, with smaller *Ln* bearing nitrides having more negative values. A discontinuity in Δ*H*_f_^°^ was further observed between larger and smaller *Ln* bearing *Ln*N compounds. Using the determined Δ*H*_f_^°^ values with literature references, Gibbs energies of formation (Δ*G*_f_^°^), oxidation (Δ*G*_ox_^°^) and hydrolysis (Δ*G*_hyd_^°^) are calculated and compared against uranium nitride (UN) in the context of UN based nuclear fuel and related spent nuclear fuel (SNF) management. The calculations indicate that the occurrence of *Ln*N phases within UN based SNF should lead to stabilisation of the structure and inhibit its oxidation.

## Abstract (web summary)

Nitride-based compounds present exceptional thermal properties for advanced material applications, but an accurate determination of their thermodynamic properties is critical for their application. Here, the authors use high-temperature oxide melt calorimetry to determine the standard enthalpies of formation (ΔHf°) for all LnN compounds (Ln = La, Ce, Pr, Nd, Sm, Eu, Gd, Tb, Dy, Ho, Er, Tm, Lu; excluding Pm), showing that ΔHf° values correlate with the ionic radius of Ln^+3^ cations, with smaller Ln-bearing nitrides exhibiting more negative values.

## Introduction

Binary lanthanide nitrides (*Ln*N) are an important class of inorganic compounds that are relevant to spintronic devices^2,3^, heterogeneous catalysis^4,5^, high-temperature applications^6,7^ and are also expected to form an important constituent within next-generation uranium nitride (UN) based spent nuclear fuel (SNF)^8–10^. The use of nitrides in nuclear energy applications arises from their improved density and higher thermal conductivity, while still providing a higher melting point over conventional oxide fuel. This has subsequently drawn considerable interest in these materials to be deployed in Generation IV fast and advanced modular reactors (AMR)^11–15^, as part of general interest in advanced fuel concepts^14,16,17^. Critically, mixed *Ln*N-UN compounds are expected to form as a predominant matrix incorporated fission product phase in UN-based SNF, which may affect its chemical stability^9,18,19^. Consequently, the understanding of their thermodynamic stability and precise oxidation behaviour is critical to their application and occurrence in nuclear and non-nuclear applications.

Thermodynamic investigations of *Ln*N and UN SNF relevant materials have primarily been achieved via computational simulation-based methods^8,20,21^. However, the success of computational methods requires precise experimental data, such as high-quality thermodynamic data, for benchmarking. For UN, its enthalpy of formation^22^ has been determined via high-temperature oxide melt calorimetry, considered the gold standard for this determination. In comparison, thermodynamic data of *Ln*N compounds remain limited. The standard enthalpy of formation (Δ*H*_f_^°^) has been measured for some *Ln*N compounds (*Ln* = La, Ce, Sm, Eu, Gd, Ho, Er, Yb) via Knudsen effusion mass spectrometry (KEMS)^22–25^.

A challenging component of *Ln*N and UN characterisation is their strong susceptibility to oxidation when in contact with atmospheric oxygen or water. This causes the materials to rapidly oxidise and compromises the measurement. It has been recently shown that under ambient conditions, UN and *Ln*N compounds oxidise under dissimilar mechanisms in which UN follows a direct O_2_ oxidation pathway, whereas *Ln*N compounds decompose through hydrolysis (H_2_O)^1^. At higher temperatures, the oxidation of *Ln*N compounds proceeds to the sesquioxide, *Ln*_2_O_3_, (*Ln* = Nd, Gd, Dy, Ho, Tm, Lu), or dioxide, *Ln*O_2_, (*Ln* = Pr, Tb)^1^. In the case of UN, at high temperatures (>200 °C), it oxidises directly via ignition to U_3_O_8_^26,27^. Despite the strong foundation of knowledge for UN, the discordant behaviour of *Ln*N compounds in terms of their oxidation chemistry demands thorough investigation.

Considering the pertinence of *Ln*N compounds to advanced material applications and also UN-based SNF, the present investigation has experimentally determined Δ*H*_f_^°^ for all *Ln*N compounds (*Ln* = La, Ce, Pr, Nd, Sm, Eu, Gd, Tb, Dy, Ho, Er, Tm, Yb and Lu, excluding Pm) using high-temperature oxide melt calorimetry. The high-temperature oxidation behaviour of *Ln*N for *Ln* = La, Ce, Sm, Eu, Er and Yb has been further characterised by thermogravimetric measurements and compared with recent measurements of *Ln* = Pr, Nd, Gd, Tb, Dy, Ho, Tm and Lu^1^. Structural characterisation of synthesised *Ln*N materials and their respective oxidation products was carried out using powder X-ray diffraction (PXRD) with Rietveld analysis. Using the Δ*H*_f_^°^ values determined and combining with literature references, the Gibbs energies of formation (Δ*G*_f_^°^), oxidation (Δ*G*_ox_^°^) and hydrolysis (Δ*G*_hyd_^°^) were calculated in comparison to UN in order to gain insight into the relative stability and behaviour of *Ln*N and UN compounds occurring in UN-based SNF. These results of the investigation are further discussed in relation to previous simulation studies and in relation to the oxidation stability of these *Ln*N and UN compounds in relation to associated SNF.

## Results and discussion

### High temperature oxidation of LnN

**Fig. 1:**
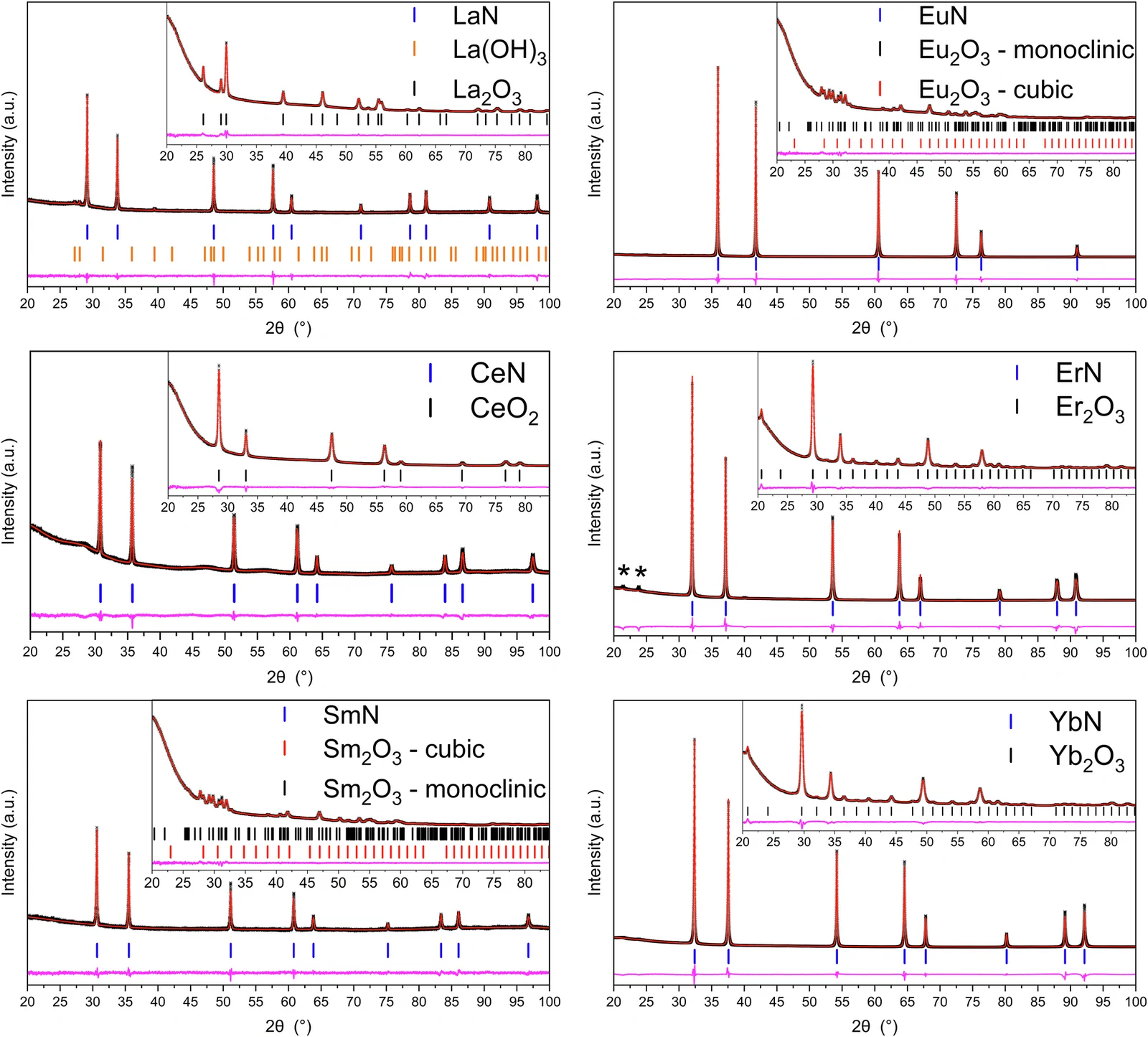
Pre- and post-oxidation PXRD comparison of investigated *Ln*N compounds. Rietveld profiles made against PXRD data of pristine *Ln*N (*Ln* = La, Ce, Sm, Eu, Er, Yb) compounds. The insets provide corresponding Rietveld profiles for the same samples oxidised in synthetic air at 900 °C. The black crosses correspond to collected data for the pre- and post-oxidised samples. The red and pink lines correspond to the calculated and difference profiles. The vertical markers correspond to the allowed reflections according to the space group of *Ln*N crystallising in cubic *Fm*$\bar{3}$*m* structure marked in blue, *Ln*_2_O_3_/*Ln*O_2_ crystallising in varying hexagonal, cubic and monoclinic structures marked in red and black, respectively (for details see Table 2), and La(OH)_3_ observed in the LaN sample crystallising in hexagonal *P*6_3_/*m* structure marked in orange. Reflexes arising from Vaseline used to fixate the powders are marked with black stars^75^.

Pristine *Ln*N powders synthesised in this work and their respective oxidation residues after heating in synthetic air at 900 °C were measured via PXRD and analysed using the Rietveld refinement method. Pristine *Ln*N samples could be consistently refined against a cubic face-centred structure in space group *Fm*$\bar{3}$*m*, isostructural with rock salt. The refinement profiles and diffractograms for pristine *Ln*N compounds (*Ln* = La, Ce, Sm, Eu, Er, Yb) are shown in Fig. 1 with refinement results summarised in Table 1, whereas the results for *Ln*N (*Ln* = Pr, Nd, Gd, Tb, Dy, Ho, Tm and Lu) have been previously reported and discussed in detail^1^.

**Table 1:** Lattice parameters determined from Rietveld refinements against PXRD data, associated fitting factors and literature lattice parameters for pristine *Ln*N (*Ln* = La, Ce, Sm, Eu, Er, Yb) phases synthesised

Compound	Structure and space group	Lattice parameter (Å)	Fitting factors (%)	Reference lattice parameter (Å)
LaN	Cubic face centred *Fm*$\bar{3}$*m*	*a* = 5.299240(20)	*R* = 2.48 *R*_*w*_ = 3.47	*a* = 5.301(2)^28^
CeN		*a* = 5.022364(20)	*R* = 1.10 *R*_*w*_ = 1.60	*a* = 5.020(2)^28^
SmN		*a* = 5.047712(26)	*R* = 2.13 *R*_*w*_ = 2.81	*a* = 5.046(2)^29^
EuN		*a* = 5.015175(7)	*R* = 3.46 *R*_*w*_ = 4.84	*a* = 5.01779(6)^30^
ErN		*a* = 4.836127(14)	*R* = 2.24 *R*_*w*_ = 3.83	*a* = 4.8481(7)^31^
YbN		*a* = 4.783650(10)	*R* = 2.32 *R*_*w*_ = 3.95	*a* = 4.775(4)^32^

Additional refinement details are provided in Supplementary Information Note 2, Table S2. Note uncertainties are reported directly from the Rietveld analysis with no correction.

It was found that the determined lattice parameters from Rietveld analysis were in agreement with structural data reported in the literature^28–32^, with small deviations attributed to non-stoichiometry in the nitrogen content of synthesised *Ln*N phases. Slight impurities of La(OH)_3_ have been observed solely in the LaN sample with 5.51 wt% and crystallising in hexagonal *P*6_3_/*m* structure, which is attributed to slight oxidation arising from contact with atmospheric water during the PXRD measurement. This water-mediated oxidation behaviour of *Ln*N compounds was recently investigated^1^, where it was shown that the *Ln*^3+^N compounds with larger and lighter *Ln*’s cations oxidised faster than the nitrides with smaller and heavier *Ln* cations.

To accurately determine Δ*H*_f_^°^ via high-temperature oxide melt calorimetry of the investigated *Ln*N compounds, their high-temperature oxidation mechanisms are required to be established. Recent studies^1^ in oxygen-containing atmospheres up to 900 °C for *Ln* = Pr, Nd, Gd, Tb, Dy, Ho, Tm and Lu show that these materials oxidise via two pathways to the respective *Ln*_2_O_3_ (*Ln* = Nd, Gd, Dy, Ho, Tm, Lu) and *Ln*O_2_ oxide compounds (*Ln* = Pr, Tb). Their reactions are described according to Eqs. (1) and (2).

1 $${LnN}+\frac{3}{4}{{\mbox{O}}}_{2}\to \frac{1}{2}{L}{{n}}_{2}{{\mbox{O}}}_{3}+\frac{1}{2}{{\mbox{N}}}_{2}$$

2 $${Ln}{{\mbox{N}}}+{{\mbox{O}}}_{2}\to {Ln}{{\mbox{O}}}_{2}+\frac{1}{2}{{\mbox{N}}}_{2}$$

With respect to the literature, *Ln*N compounds for *Ln* = La, Ce, Sm, Eu, Er, Yb have not been investigated under similar conditions to those of the present investigation and necessary for a calorimetric study. It has been previously suggested that ErN forms Er_2_O_3_ under oxidising conditions, but details are lacking on the precise temperature at which the transformation occurs^33^. To address this, coupled TGA-DSC measurements were performed on these *Ln*N compounds (*Ln* = La, Ce, Sm, Eu, Er, Yb), where powder samples were heated to 900 °C in synthetic air, and the residues after oxidation were characterised via PXRD. Rietveld profiles made against the PXRD data are presented in the inset to their respective *Ln*N phases in Fig. 1. The analysis revealed that all *Ln*N compounds investigated follow an oxidation pathway as given by Eqs. (1) or (2). Refined lattice parameters of the oxidised *Ln*N phases are summarised in Table 2 and are in agreement with the lattice parameters reported in the literature.

**Table 2:** Summary of determined crystalline phases arising from the oxidation of LnN (Ln = La, Ce, Sm, Eu, Er and Yb) in air at 900 °C via Rietveld analysis and their respective literature lattice parameters

Compound	Oxidised phase	Structure and space group	Lattice parameters (Å)	Fitting factors (%)	Literature lattice parameter (Å)
LaN	La_2_O_3_	*P*6_3_/*mmc* (194)	*a* = 3.9377(2) *c* = 6.1388(2)	*R* = 1.70 *R*_*w*_ = 2.57	*a* = 3.9381(3) *c* = 6.1361(6)^66^
CeN	CeO_2_	*Fm*$\bar{3}$*m* (225)	*a* = 5.4142(1)	*R* = 2.18 *R*_*w*_ = 3.31	*a* = 5.4178(1)^67^
SmN	Sm_2_O_3_	*C*2*/m* (12)	*a* = 14.175(2) *b* = 3.6331(2) *c* = 8.8574(9) *β* = 100.189(5)	*R* = 1.34 *R*_*w*_ = 2.02	*a* = 14.1975(9) *b* = 3.6273(3) *c* = 8.8561(5) *β* = 99.986(5)^68^
	Sm_2_O_3_	*Ia*$\bar{3}$ (206)	*a* = 10.931(1)		*a* = 10.9295(5)^69^
EuN	Eu_2_O_3_	*C*2*/m* (12)	*a* = 14.110(2) *b* = 3.6061(2) *c* = 8.8088(9) *β* = 100.161(5)	*R* = 1.39 *R*_*w*_ = 2.08	*a* = 14.1105(2) *b* = 3.6021(1) *c* = 8.8080(2) *β* = 100.037(1)^70^
	Eu_2_O_3_	*Ia*$\bar{3}$ (206)	*a* = 10.869(1)		*a* = 10.86831(24)^71^
ErN	Er_2_O_3_	*Ia*$\bar{3}$ (206)	*a* = 10.4417(2)	*R* = 1.89 *R*_*w*_ = 2.62	*a* = 10.5486(9)^72^
YbN	Yb_2_O_3_	*Ia*$\bar{3}$ (206)	*a* = 10.4378(2)	*R* = 2.34 *R*_*w*_ = 3.19	*a* = 10.4281(2)^73^

Additional refinement results provided by the Rietveld software GSAS-II are shown in Supplementary Information Note 2, Table S2. Note that uncertainties are reported directly from the Rietveld analysis with no correction.

**Fig. 2:**
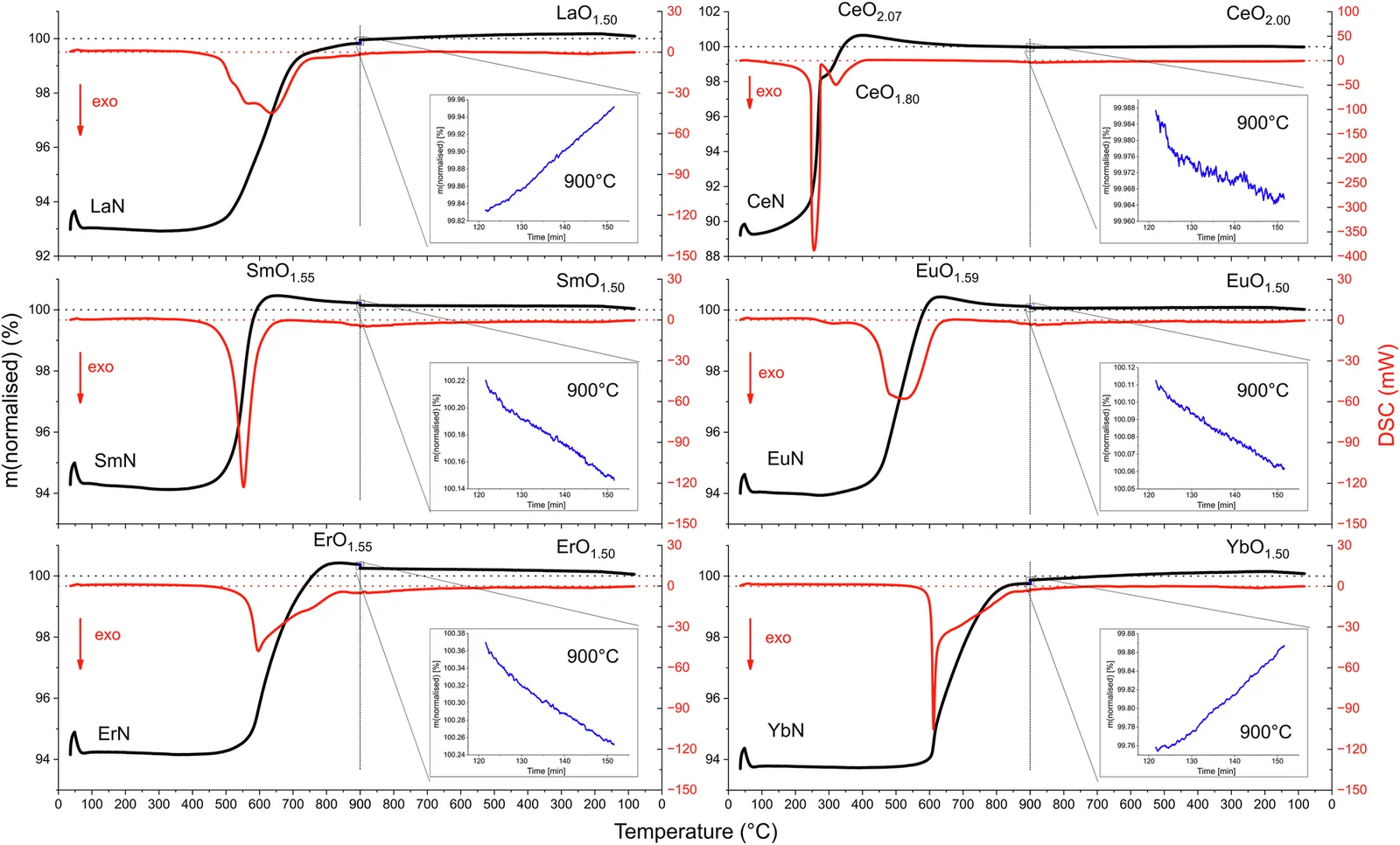
TGA profiles for investigated *Ln*N compounds. Combined heating and cooling black TGA- and red DSC curves of LaN, CeN. SmN, EuN, ErN and YbN heated to 900 °C in air with inset blue TGA curve of plateau performed at 900 °C.

Simultaneous TGA and DSC measurements were performed from room temperature to 900 °C under flowing air conditions for the *Ln*N compounds with *Ln* = La, Ce, Sm, Eu, Er and Yb. These were performed to ensure consistency with previous measurements taken under similar conditions^1^. The resulting profiles are displayed in Fig. 2, where the TGA data were normalised against the fully oxidised *Ln*N compounds that were recovered after cooling. This has been performed since the pristine *Ln*N exhibits varying stoichiometries at room temperature, compromising sum formula calculations based on the weight change. All determined and calculated TGA and DSC results are summarised in Table 3. It was found that all *Ln*N compounds oxidise in a direct oxygen-mediated reaction following the reactions shown in Eqs. (1) and (2), consistent with previous investigation^1^. However, it must be noted that the oxidation of CeN proceeds via an observable intermediate oxide, whose composition could be determined approximately as CeO_1.80_. The formation of a distinct intermediate can be attributed to the intrinsic tendency of Ce to form mixed-valence oxidation states of +3 and +4 in oxide form, which is also observed for PrN and TbN^1^.

**Table 3:** Summary of TGA-DSC determined onset temperatures *T*_onset_ and temperatures of the DSC maxima *T*_DSC_, experimental and theoretical mass changes and via PXRD analysis determined oxidation products of *Ln*N (*Ln* = La, Ce, Sm, Eu, Er, Yb) oxidised in air at 900 °C

Compound	*T*_onset_ (°C) *	*T*_DSC_ (°C)	Δm_total_ (%)	Δm_theo_ (%) (oxidation product)	Oxidation product
LaN	495.9	566.3 636.41	6.97	6.52 (La_2_O_3_)	La_2_O_3_
CeN	175.0	255.7 320.7	11.38	11.66 (CeO_2_)	CeO_2_
SmN	487.3	552.6	6.14	6.07 (Sm_2_O_3_)	Sm_2_O_3_
EuN	420.5	526.3	6.38	6.01 (Eu_2_O_3_)	Eu_2_O_3_
ErN	551.7	596.4	6.18	5.50 (Er_2_O_3_)	Er_2_O_3_
YbN	603.4	612.1	6.21	5.33 (Yb_2_O_3_)	Yb_2_O_3_

All temperature values have an uncertainty of σ = ± 1 °C given by the used thermocouple type S.

^*^Defined as 5% of total mass change of the nitride to oxide reaction.

### High temperature oxide melt calorimetry

High-temperature oxide melt calorimetric measurements were conducted on all *Ln*N (*Ln* = La, Ce, Pr, Nd, Sm, Eu, Gd, Tb, Dy, Ho, Er, Tm, Yb, Lu; excluding Pm) compounds to determine their standard enthalpies of formation Δ*H*_f_^°^. Two thermochemical cycles which are provided in Table 4 and Table 5 have been developed based on the oxidation pathways shown in Eqs. (1) and (2), which were used to calculate Δ*H*_f_^°^ based on the measured dissolution enthalpies, (Δ*H*_ds_) of *Ln*N in sodium molybdate at 973 K. Experimental Δ*H*_ds_ data are summarised in Supplementary Information Note 3, Table S3 together with literature enthalpies of the corresponding *Ln* oxides used for calculation. The resulting Δ*H*_f_^°^ values of the *Ln*N compounds examined in this investigation are summarised in Table 6 with their associated standard error. Error propagation with the two standard errors of the dissolution enthalpies 2σ(Δ*H*_ds_) (summarised in Supplementary Information Note 3, and Table S3) and standard errors of the published literature data (summarised in Supplementary Information Note 3, and Table S3) has been performed for all datasets. The dataset of CeN was corrected for the TiO_2_ encapsulation used to protect the sample against premature ignition using appropriate literature values^34^.

**Table 4:** Thermochemical cycle used for calculation of Δ*H*_f_^°^ for the *Ln*N compounds where *Ln* = La, Nd, Sm, Eu, Gd, Dy, Ho, Er, Tm, Yb and Lu oxidise to the respective *Ln*_2_O_3_, based on drop-solution calorimetric data of the *Ln*N compounds in sodium molybdate at 973 K

Reaction		Δ*H* (kJ mol^-1^)
(1)	*Ln*N_(s, 298 K)_ + $\frac{3}{2}$ O_2(g, 973 K)_ → $\frac{1}{2}$ *Ln*_2_O_3(sln, 973 K)_ + $\frac{1}{2}$ N_2(g, 973 K)_	Δ*H*_1_ = Δ*H*_ds_ *
(2)	*Ln*_2_O_3(s, 298 K)_ → *Ln*_2_O_3(s, 973 K)_	Δ*H*_2_ *
(3)	O_2(g, 298 K)_ → O_2(g, 973 K)_	Δ*H*_3_ = 21.74(2)^53^
(4)	N_2(g, 298 K)_ → N_2(g, 973 K)_	Δ*H*_4_ = 20.65^74^
(5)	2 *Ln*_(s, 298 K)_ + $\frac{3}{2}$ O_2(g, 298 K)_ → *Ln*_2_O_3(s, 298 K)_	Δ*H*_5_ = Δ*H*_f_^°^(*Ln*_2_O_3_) *
**Enthalpy of Oxidation** ***Ln*N** → ***Ln***_**2**_**O**_**3**_		
(6)	*Ln*N_(s, 298 K)_ + $\frac{3}{2}$ O_2(g, 298 K)_ → $\frac{1}{2}$ *Ln*_2_O_3(s, 298 K)_ + $\frac{1}{2}$ N_2(g, 298 K)_	
	Δ*H*_ox_ = Δ*H*_ds_ + $\frac{3}{2}$ Δ*H*_3_ - $\frac{1}{2}$ Δ*H*_2_ - $\frac{1}{2}$ Δ*H*_4_	
**Enthalpy of Formation** ***Ln*N**		
(7)	*Ln*_(s, 298 K)_ + $\frac{1}{2}$ N_2(g, 298 K)_ → *Ln*N_(s, 298 K)_	
	Δ*H*_f_^°^(*Ln*N) = - Δ*H*_ox_ + $\frac{1}{2}$ Δ*H*_5_	

The summarised values of dissolution enthalpies Δ*H*_ds_ and additional enthalpies required are provided in Supplementary Information Note 3, Table S3. Resulting standard enthalpy of formation of *Ln*N are summarised in Table 6.

s = solid, g = gaseous, sln = solution.

^*^Values summarised in Supplementary Information Note 3, Table S3.

**Table 5:** Thermochemical cycle used for calculation of Δ*H*_f_^°^ for the *Ln*N compounds where *Ln* = Ce, Pr, Tb oxidise to the respective *Ln*O_2_, based on drop-solution calorimetric data of *Ln*N compounds in sodium molybdate at 973 K

Reaction		Δ*H* (kJ mol^-1^)
(1)	*Ln*N_(s, 298 K)_ + O_2(g, 973 K)_ → *Ln*O_2(sln, 973 K)_ + $\frac{1}{2}$ N_2(g, 973 K)_	Δ*H*_1_ = Δ*H*_ds_ *
(2)	*Ln*O_2(s, 298 K)_ → *Ln*O_2(s, 973 K)_	Δ*H*_2_ *
(3)	O_2(g, 298 K)_ → O_2(g, 973 K)_	Δ*H*_3_ = 21.74(2)^53^
(4)	N_2(g, 298 K)_ → N_2(g, 973 K)_	Δ*H*_4_ = 20.65^74^
(5)	*Ln*_(s, 298 K)_ + O_2(g, 298 K)_ → *Ln*O_2(s, 298 K)_	Δ*H*_5_ = Δ*H*_f_^°^(*Ln*O_2_) *
**Enthalpy of Oxidation** ***Ln*N** → ***Ln*O**_**2**_		
(6)	*Ln*N_(s, 298 K)_ + O_2(g, 298 K)_ → *Ln*O_2(s, 298 K)_ + $\frac{1}{2}$ N_2(g, 298 K)_	
	Δ*H*_ox_ = Δ*H*_ds_ + Δ*H*_3_ - Δ*H*_2_ - $\frac{1}{2}$ Δ*H*_4_	
**Enthalpy of Formation** ***Ln*N**		
(7)	*Ln*_(s, 298 K)_ + $\frac{1}{2}$ N_2(g, 298 K)_ → *Ln*N_(s, 298 K)_	
	Δ*H*_f_^°^(*Ln*N) = - Δ*H*_ox_ + Δ*H*_5_	

The summarised values of dissolution enthalpies, Δ*H*_ds_, and additional enthalpies required are provided in Supplementary Information Note 3, Table S3. Resulting standard enthalpy of formation of *Ln*N are summarised in Table 6.

s = solid, g = gaseous, sln = solution.

^*^Values summarised in Supplementary Information Note 3, Table S3.

**Table 6:** Standard enthalpy of formation Δ*H*_f_^°^ for *Ln*N compounds

Compound	Δ*H*_f_^°^ (kJ mol^-1^)	σ (Δ*H*_f_^°^) (kJ mol^-1^)
LaN	−316.92	13.1
CeN	−388.35	8.1
PrN	−321.24	15.4
NdN	−375.22	9.0
SmN	−385.98	11.5
EuN	−328.28	7.8
GdN	−368.28	19.4
TbN	−347.28	11.0
DyN	−489.26	12.9
HoN	−517.75	7.3
ErN	−566.98	16.7
TmN	−489.82	11.7
YbN	−529.08	8.1
LuN	−549.71	5.6

Enthalpies are based on performed high-temperature oxide melt calorimetry experiments and their respective two standard errors σ (Δ*H*_f_^°^).

**Fig. 3:**
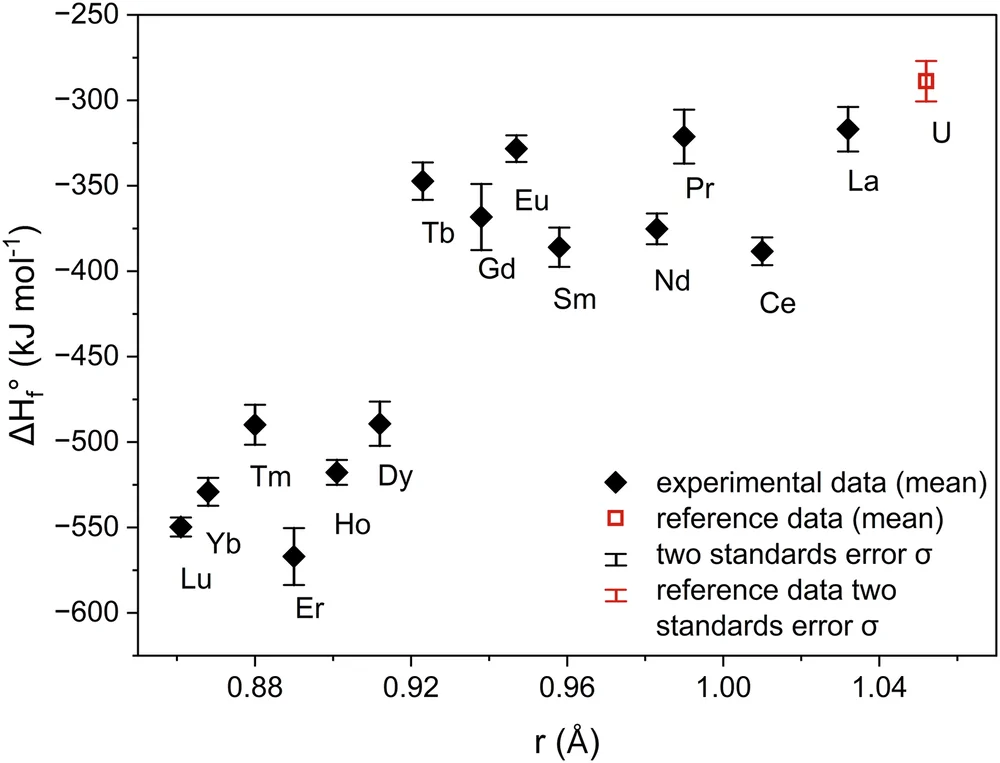
Formation Enthalpies of *Ln*N compounds compared against UN. Experimentally determined Δ*H*_f_^°^ of *Ln*N (*Ln* = La, Ce, Pr, Nd, Sm, Eu, Gd, Tb, Dy, Ho, Er, Tm, Yb and Lu) plotted against the ionic radii, r, of the *Ln*^3+^ cation in 6-fold coordination. The literature Δ*H*_f_^°^ of UN determined by the same methodology is plotted for comparison^22^. Both the experimental and reference data points represent the arithmetic mean with error bars that represent ±2 standard error of the mean.

Figure 3 provides the determined Δ*H*_f_^°^ of all *Ln*N compounds plotted against the ionic radii of *Ln*^3+^ cations in sixfold coordination^35^. The Figure pertinently shows that the *Ln*N compounds that possess smaller and heavier *Ln* cations exhibit lower Δ*H*_f_^°^, whereas those with larger and lighter *Ln* cations show less negative values. The data of heavy *Ln*’s contrasts significantly with previously determined Δ*H*_f_^°^ values based on KEMS investigation^23–25^, which suggests largely consistent values of Δ*H*_f_^°^ in the range of −300 to −350 kJ mol^−1^ and is plotted for comparison in Supplementary Information Note 3, and Fig. S2.

Additionally, the determined Δ*H*_f_^°^ values for the *Ln*N compounds exhibit a discontinuity in the enthalpy trend between TbN and DyN, separating the series into lighter *Ln* (La, Ce, Pr, Sm, Eu, Gd, Tb) with higher Δ*H*_f_^°^, and heavier *Ln* (Dy, Ho, Er, Tm, Yb, Lu) with lower Δ*H*_f_^°^. This break is consistent with the half-filling of the 4*f* orbitals, due to which *Ln*N containing heavier *Ln* cations exhibit higher stability compared to *Ln*N containing lighter *Ln* cations. However, Tb is a notable exception to the trend in Fig. 3. Such a deviation could be related to a potential covalent contribution to the bond in TbN, attributable to mixed ionic and covalent bonding behaviour within nitride materials^36^. However, from the analysis of this investigation, this cannot be precisely elucidated.

Figure 3 additionally compares the Δ*H*_f_^°^ for the *Ln*N compounds with that of UN (Δ*H*_f_^°^ = −288.8 kJ mol^−1^), which was determined by the same methodology on the same experimental instrument^22^. It shows that UN exhibits a less negative enthalpy of formation than all *Ln*N, which aligns well with the observed trend that larger cationic radii^35^ of +3 cations in sixfold coordination environment correspond to less negative Δ*H*_f_^°^.

### The Gibbs energy of formation and oxidation stability for LnN and UN compounds in the context of UN based spent nuclear fuel

To summarise thus far, the present investigation has systematically and accurately determined Δ*H*_f_^°^ and high-temperature oxidation chemical reactions of all *Ln*N compounds except for Pm. Considering the relevance of these phases to UN-based SNF, it is salient to discuss their occurrence and associated thermodynamic chemistry with the results of this investigation considered. In UN-based SNF, *Ln*N compounds are expected to form as a major group of fission products that are readily miscible within the UN matrix owing to their shared crystal structure^1,37^ and demonstrated solid solution behaviour^9,18,19^. The degree and type of solid solution, for instance, constituents and amounts, significantly impact the properties of UN. For instance, it has been shown for Ce- and Gd-doped UN solid solutions that increasing the amount of Ce-content reduces oxidation resistivity at high temperatures^38^; whereas increasing Gd-content reduces thermal conductivity^39^. At the same time, computational studies of Zr- and Y-doped UN indicate oxidation resistance^40^. In situ steam experiments indicate that Zr-doped UN oxidation resistance also occurs^41^. Although from the literature, a definitive answer as to the stability of UN materials from doping is not present, it is clear that the *Ln* incorporation affects the stability of UN. Such effects are well known for UO_2_-based SNF^42^ with a strong thermodynamic description behind it^42,43^, whereby the incorporation of *Ln* improves resistance to oxidation. To assess analogous behaviour in UN systems, a thermodynamic framework is required. In this regard, the Gibbs energy of formation as nitrides, Δ*G*_f_^°^, the Gibbs energy of oxidation to oxide state, Δ*G*_ox_^°^, and the Gibbs energy of hydrolysis to hydroxide/oxide state, Δ*G*_hyd_^°^, are evaluated using the enthalpic data determined from this investigation. Combined with reference entropy values from literature^44–54^ Δ*G*_f_^°^, Δ*G*_ox_^°^ and Δ*G*_hyd_^°^ can be calculated via the Gibbs equation for all investigated *Ln*N compounds and also in reference to UN when assuming conditions of RT oxidation.

The Gibbs equation, as shown in Eq. (3), uses the reaction enthalpy, Δ*H*_rct_^°^ and reaction entropy, Δ*S*_rct_^°^, of a chemical reaction, which are determined via Hess’s law shown in Eqs. (4) and (5). This is achieved by subtracting the sum of enthalpies of formation or standard entropies of the reaction products from the sum of the reactants, respectively.

3 $${\Delta G}^{ \circ }={\triangle H}_{{{\rm{rct}}}}^{ \circ }-{{{\rm{T}}}\triangle S}_{{{\rm{rct}}}}^{ \circ }$$

4 $${{\Delta}{H}}_{{\rm{rct}}}^{\circ} = \sum \nu {\left({{\Delta}{H}}_{{\mbox{f}}}^{\circ}\right)}_{{\rm{products}}}- \sum \nu {\left({\Delta}{H}_{{\rm{f}}}^{\circ}\right)}_{{\rm{educts}}}$$

5 $${\Delta}{S}_{{\mbox{rct}}}^{ \circ } =\sum \nu {S^{ \circ } }_{{\mbox{products}}}-\sum \nu {S^{ \circ } }_{{\mbox{educts}}}$$

To calculate Δ*G*_f_^°^, the formation reactions of *Ln*N and UN compounds are required, which are provided in Eqs. (6) and (7). Specifically for the formation of materials as in the present study, Δ*H*_rct,f_^°^ can be replaced by the formation enthalpies determined for *Ln*N and literature data on UN^22^. Reaction formation entropies Δ*S*_rct,f_^°^ are calculated using literature values for all compounds, which are summarised in Supplementary Information Note 4 Table S4. Since the standard entropies *S*^°^ on pure *Ln*N materials are scarcely known, the available values for LaN and CeN summarised in Supplementary Information Note 4 Table S4 were used as a replacement for all *Ln*N.

6 $${Ln}+\frac{1}{2}{{\mbox{N}}}_{2}\to {Ln}{{\mbox{N}}}$$

7 $${{\mbox{U}}}+\frac{1}{2}{{\mbox{N}}}_{2}\to {{\mbox{UN}}}$$

In order to describe the oxidation stability of *Ln*N and UN compounds via Δ*G*_ox_^°^, a similar thermodynamic methodology is applied. It is assumed that the oxidation occurs in a dry oxygen atmosphere for simplicity, although it was previously shown that *Ln*N compounds react preferably with atmospheric water when contacting with air at room temperature^1^. Given this assumption, the oxidation of *Ln*N is described via the pathways in Eqs. (1) and (2). The oxidation of UN at 298.15 K can be described in a simplified form from literature observations^22,55,56^ as shown in Eq. (8).

8 $${\mbox{UN}}+{{\mbox{O}}}_{2}\to {\mbox{U}}{{\mbox{O}}}_{2}+\frac{1}{2}{{\mbox{N}}}_{2}$$

Δ*H*_f_^°^ values used to calculate the reaction enthalpy of oxidation Δ*H*_rct,ox_^°^ are summarised in Tables 4 to 6 and Supplementary Information Note 3, Table S3. The standard entropies used to calculate the reaction entropy of oxidation Δ*S*_rct,ox_^°^ for *Ln*N and UN are summarised in Supplementary Information Note 4, Table S4. As described earlier, the standard entropies *S*^°^ on pure *Ln*N materials are scarcely known; consequently, the available values for LaN and CeN are summarised in Supplementary Information Note 4 Table S4 were used as a replacement for all *Ln*N.

Previously conducted oxidation experiments of *Ln*N in air at RT conditions have revealed that these compounds react via a water-mediated oxidation pathway, resulting in the formation of the complementary *Ln*(OH)_3_ as shown in Eq. (9)^1^. Thus, to establish a thermodynamic framework of their oxidation behaviour, the hydrolysis of *Ln*N and UN via Δ*G*_hyd_^°^ must also be considered. In this regard, the hydrolysis of UN can be described with respect to the literature as shown Eq. (10)^57–59^.

9 $$\,{Ln}{{\mbox{N}}}+3\,{{\mbox{H}}}_{2}{{\mbox{O}}}\to {Ln}{\left({\mbox{OH}}\right)}_{3}+{{\mbox{N}}}{{\mbox{H}}}_{3}$$

10 $${{\mbox{UN}}}+2{{\mbox{H}}}_{2}{{\mbox{O}}}\to {{\mbox{U}}}{{\mbox{O}}}_{2}+{{\mbox{N}}}{{\mbox{H}}}_{3}+\frac{1}{2}{{\mbox{H}}}_{2}$$

All hydrolysis calculations were performed assuming that water participating in the hydrolysis was in the gaseous state. The reaction enthalpy of hydrolysis Δ*H*_rct,hyd_^°^ was derived using the formation enthalpies summarised in Tables 4 to 6 and Supplementary Information Note 3, Table S3. The standard entropies used to calculate the reaction entropy of hydrolysis Δ*S*_rct,hyd_^°^ are summarised in Supplementary Information Note 4, Table S4. Once again, given that standard entropies *S*^°^ on pure *Ln*N materials are scarcely known, entropy values for LaN and CeN were used as a replacement for all *Ln*N. Additionally, *S*^°^ for Lu(OH)_3_ was replaced by the available literature *S*^°^ of Tm(OH)_3_^60^. All thermodynamic data that were calculated via the methodology described are summarised in Table 7 and Supplementary Information Note 4, Table S5.

**Table 7:** Gibb’s energies of formation Δ*G*_f_^°^ for *Ln*N and UN, their oxidation Δ*G*_ox_^°^ and their hydrolysis Δ*G*_hyd_^°^ at 298.15 K

Compound	Δ*G*_f_^°^ (kJ mol^-1^)	r_ionic_ (*Ln*/U in oxide) (Å)^35^	Δ*G*_ox_^°^ (kJ mol^-1^)	r_ionic_ (*Ln*/U in hydoxide) (Å)^35^	Δ*G*_hyd_^°^ (kJ mol^-1^)
LaN	−303.90	1.16	−573.31	1.16	−330.12
CeN	−371.59	0.87	−686.21	1.143	−262.14
PrN	−303.15	0.85	−622.64	1.126	−327.63
NdN	−357.97	1.109	−530.44	1.109	−274.91
SmN	−369.21	0.958	−523.42	1.079	−253.94
EuN	−309.03	0.947	−493.76	1.066	−222.37
GdN	−351.97	0.938	−540.43	1.053	−274.18
TbN	−329.37	0.76	−605.45	1.04	−301.60
DyN	−470.88	0.912	−440.22	1.027	−173.80
HoN	−499.33	0.901	−422.66	1.015	−147.93
ErN	−549.11	0.89	−381.35	1.004	−99.67
TmN	−471.71	0.88	−451.10	0.994	−164.80
YbN	−515.19	0.868	−373.46	0.985	−97.59
LuN	−538.47	0.861	−380.63	0.861	−110.81
UN	−283.08	1.00	−779.34	1.00*	−327.17

Calculated using Eqs. (3) to (5), based on the reactions shown in Eqs. (1), (2), and (6) to Eq. (10). Additionally, literature ionic radii^35^ of *Ln*/U cations in the oxide/hydroxide materials after oxidation/hydrolysis used to plot the Gibbs energies in Fig. 5 are summarised. Note that the respective ionic radii of the cations were chosen according to their coordination numbers and oxidation states (*Ln*^4+^ for *Ln* = Ce, Pr, Tb and U^4+^) in the resulting oxidation product.

^*^ Hydrolysis reaction product = UO_2_, see Eq. (10).

**Fig. 4:**
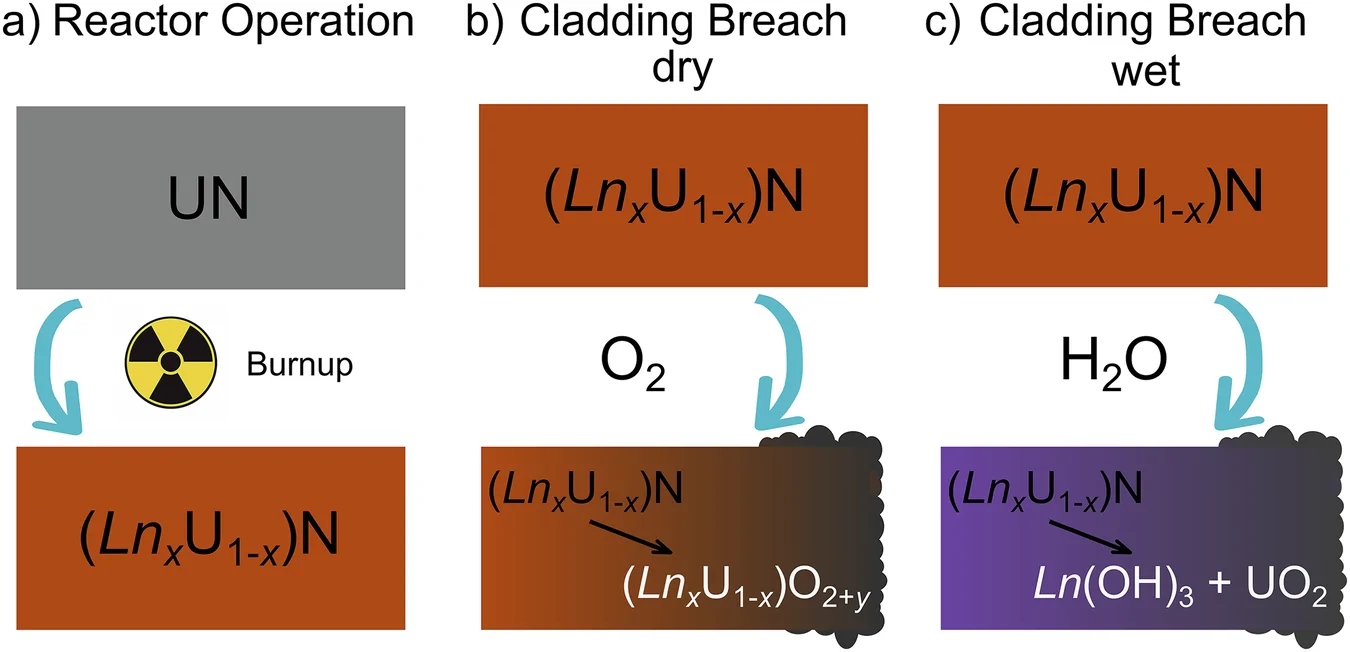
Stability scenarios for UN based SNF. Schematic illustration of ideal stability case scenarios comparing (**a**) formation of *Ln*N against UN in UN based SNF during irradiation, (**b**) oxidation of *Ln*N against UN in a cladding breach scenario involving oxygen-fuel contact and (**c**) hydrolysis of *Ln*N against UN in a cladding breach scenario involving water-fuel contact under RT conditions.

**Fig. 5:**
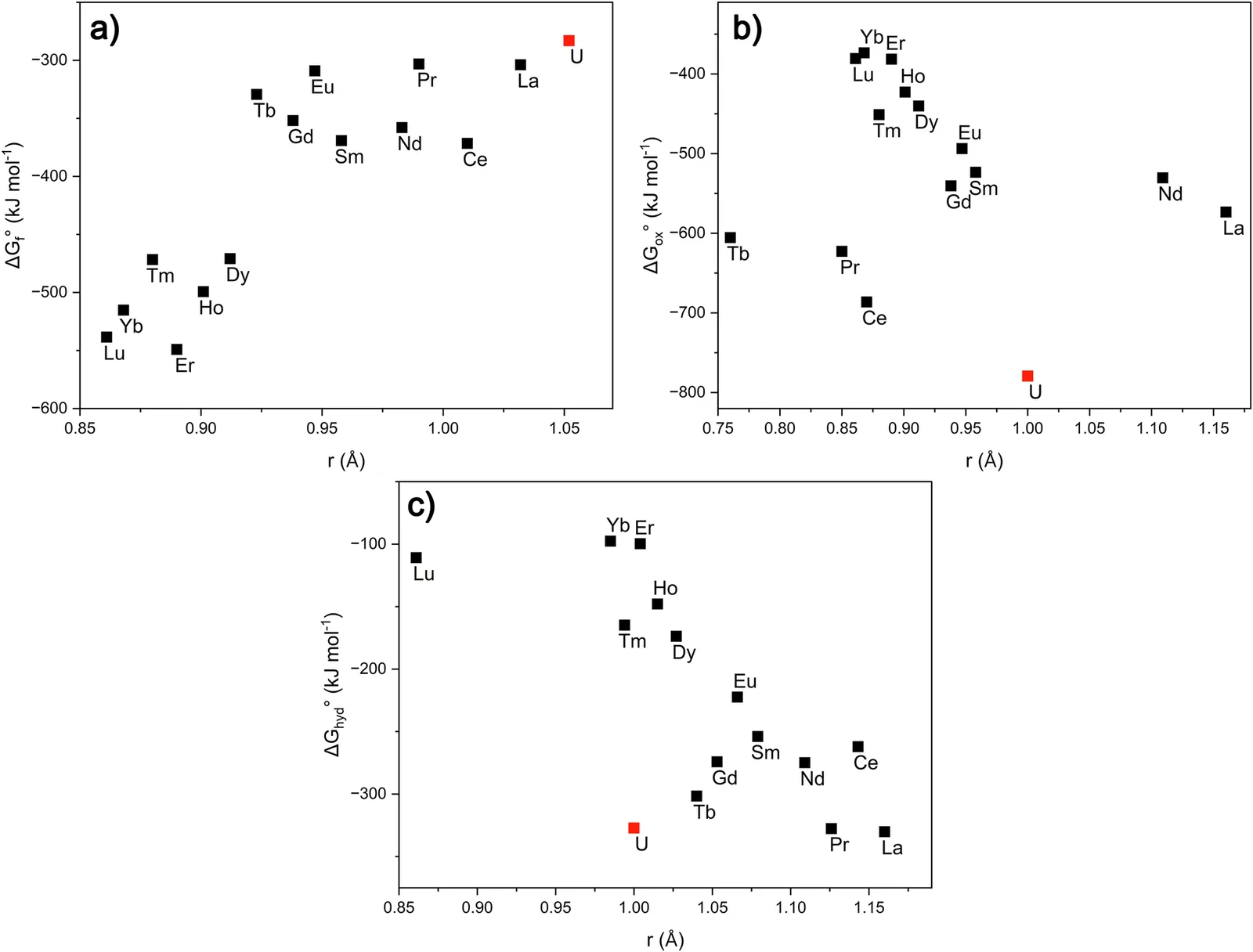
Comparison of Gibbs energies for formation Δ*G*_f_^°^, oxidation Δ*G*_ox_^°^ and hydrolysis Δ*G*_hyd_^°^ of investigated *Ln*N compounds against UN. Gibbs energies of (**a**) formation Δ*G*_f_^°^ for *Ln*N and UN, (**b**) oxidation Δ*G*_ox_^°^ for *Ln*/U oxides (*Ln*_2_O_3_ for *Ln* = La, Nd, Sm, Eu, Gd, Dy, Ho, Er, Tm, Lu; *Ln*O_2_ for *Ln* = Ce, Pr, Tb) and (**c**) hydrolysis Δ*G*_hyd_^°^ for *Ln*(OH)_3_/UO_2_ plotted against the ionic radii^35^ of the *Ln*/U cation in the respective compound. All Gibbs energies are calculated for 298.15 K as shown in Eqs. (3) to (5), based on the chemical reactions described for the formation of *Ln*N and UN see Eqs. (6) and (7), as well as their oxidation in Eqs. (1), (2), (8) and Eqs. (9) to (10). The Gibbs energies and literature ionic radii of varying oxide/hydroxide structures used for plotting are summarised in Table 7. Note that the respective ionic radii of the cations were chosen according to their coordination numbers and oxidation states (*Ln*^4+^ for *Ln* = Ce, Pr, Tb and U^4+^) in the resulting oxidation product.

Comparison of the calculated Gibbs energies allows evaluation of the aforementioned stability case scenarios: (a) relative stability of *Ln*N compounds formed within and compared to UN within UN-based SNF, (b) the subsequent stability of UN SNF against oxidation and (c) the stability of UN SNF against hydrolysis, such as what may occur during an unexpected cladding breach. These scenarios are illustrated in Fig. 4a–c, and the calculated Δ*G*_f_^°^, Δ*G*_ox_^°^ and Δ*G*_hyd_^°^ values are plotted as a function of ionic radii in Fig. 5a–c, respectively. Note that the schemes assume ideal reaction scenarios, without passivation of phases.

Figure 5a compares calculated Δ*G*_f_^°^ of *Ln*N with UN by plotting them against the ionic radii of the *Ln*^3+^/U^3+^ cation in a sixfold coordination environment in the isostructural nitrides crystallising in *Fm*$\bar{3}$*m* structure. At 298.15 K, all *Ln*N exhibit comparable more negative Δ*G*_f_^°^ values than UN, indicating thermodynamically favourable formation of nitrides as described in Eqs. (6) and (7). Consequently, it can be inferred that all *Ln*N compounds examined exhibit a higher stability than UN, implying that a solid solution with UN in UN-based SNF will be stabilised by the presence of secondary fission product *Ln*N phases under the given conditions. It is, however, stressed that this investigation is directed at end member compositions where solid solution measurements are necessary to test the veracity of this postulate. Additionally, *Ln*N compounds with heavier and smaller *Ln* cations exhibit comparable lower Δ*G*_f_^°^ values than *Ln*N compounds with lighter and larger *Ln* cations, which are more closely related to the Δ*G*_f_^°^ calculated for UN. Hence, *Ln*N fission product phases with heavier and smaller *Ln* cations are expected to provide a greater stabilisation effect on the UN matrix. These results also suggest that burnup of UN fuel, through the accumulation of *Ln-*based fission products that are lattice matrix soluble, will affect the thermodynamic stability of UN and suggest an enhancement in the energetic stability of the UN-based SNF matrix.

Scenario b) compares the oxidation of *Ln*N and UN as described in Eqs. (1), (2), and (8) shift the focus to the decomposition of the nitrides in the presence of oxygen. A measure to describe this chemical behaviour is presented by the calculated Δ*G*_ox_^°^ plotted in Fig. 5b against the ionic radii of the *Ln*/U cations in the oxide compounds. Unlike Scenario a), the comparison is more complex as the oxides are not isostructural; consequently Table 5 additionally provides the literature ionic radii^35^ values of *Ln*/U cations in their respective oxide structures, considered for plotting Δ*G*_ox_^°^ against^35^. From Fig. 5b, it is observed that all *Ln*N compounds have less negative Δ*G*_ox_^°^ values than UN, specifically under the given conditions, equivalent to a preferred oxidation of UN over *Ln*N. This result implies that a solid solution of UN with *Ln*N will be stabilised against oxidation, consistent with previous findings^1^. Consistent with Scenario a), heavier *Ln* cations provide an increased stabilising effect, indicating that increasing fission product accumulation from higher burnup enhances the oxidation resistance of UN-based SNF. It should be emphasized that these implications depend on the stated assumptions, particularly variation in temperature and composition of the exposed atmosphere, both of which can have significant effects upon Δ*G*_ox_^°^. Nevertheless, the results of the investigation are consistent with experimental investigations on the stability of doped UN materials^40,41^, pointing towards increased thermodynamic and oxidation stability in the occurrence of *Ln*-doped UN.

Recent investigations have shown that in the presence of air containing both atmospheric oxygen and water, *Ln*N compounds oxidise via water to form the respective *Ln*(OH)_3_ instead of oxides. Therefore, the hydrolysis reaction must also be considered to establish a thermodynamic perspective on the oxidation behaviour of these compounds. Scenario c) compares the hydrolysis of *Ln*N against UN, as described by Eqs. (9) and (10). As a measure for comparison, the Gibbs energies of hydrolysis Δ*G*_hyd_^°^ are presented and plotted in Fig. 5c against the literature ionic radii^35^ of *Ln*/U cations in the respective *Ln*(OH)_3_ and UO_2_. Note that it was shown that *Ln*(OH)_3_ resulting from *Ln*N air oxidation forms crystalline structures only for *Ln* = Pr, Nd, while the *Ln* = Gd, Tb, Dy, Ho, Tm, Lu were observed to form amorphous structures^1^. For the present comparison of Δ*G*_hyd_^°^ it was assumed that all *Ln*(OH)_3_ will be crystallising isostructural in the hexagonal *P*6_3_/*m* (#176) space group, as observed for Pr(OH)_3_ and Nd(OH)_3_^1^, in order to choose the ionic radii^35^ from literature for the respective *Ln* cation to plot against. From Fig. 5c, it becomes apparent that Δ*G*_hyd_^°^ for *Ln*N is comparably higher than for UN, which, given the stated conditions, is equivalent to a preferred hydrolysis of UN over *Ln*N. Similar to the oxidation scenario b), this implies that *Ln*N will have a stabilising effect on the UN matrix. Consistent with scenarios a) and b), the results imply that a higher content of fission products, particularly with heavier *Ln* cations, will increase the hydrolysis stability of UN SNF. This effect correlates with the burnup of the fuel, indicating that a higher burnup increases the hydrolysis resistance. Notably, these implications are strongly based on the stated assumptions and are influenced by the temperature and exact composition of the exposed atmosphere, which can significantly affect Δ*G*_hyd_^°^.

## Conclusions

High-temperature oxide melt calorimetry experiments were performed to determine Δ*H*_f_^°^ for all *Ln*N compounds (*Ln* = La, Ce, Pr, Nd, Sm, Eu, Gd, Tb, Dy, Ho, Er, Tm, Yb, Lu; excluding Pm) which are isostructural. A clear trend is observed whereby the Δ*H*_f_^°^ of *Ln*N compounds with larger and lighter *Ln* exhibits more negative values than those of *Ln*N with smaller and heavier *Ln*. A distinct discontinuity observed in Δ*H*_f_^°^ further highlights the role of 4 *f* electrons in governing thermodynamic stability. Comparison with isostructural UN, for which Δ*H*_f_^°^ was determined using the same methodology previously^22^, further supports these trends. Due to the relevance of UN and *Ln*N compounds to UN-based SNF, Δ*G*_f_^°^, Δ*G*_ox_^°^ and Δ*G*_hyd_^°^ were derived based on the results of this work and literature data. These analyses suggest that *Ln*N compounds exhibit generally higher chemical stability compared to UN, and the presence of potential UN-*Ln*N solid-solution phases within SNF will enhance structural stability and oxidation resistance during irradiated and air-cladding breach scenarios. Moreover, the work provides critical thermodynamic data for *Ln*N systems, advancing the understanding of UN-based SNF, and offering broader insights into general nitride materials that can be found in advanced technology applications like fusion energy and thermoelectric in which *Ln*N compounds play a fundamental role.

## Methods

### Synthesis

The synthesis and utilisation of the *Ln*N powders used in the investigation are derived from a previous study^1^. A direct nitridation approach was used, in which fine pieces of lanthanide metals (Hunan Rare Earth Metals Materials Co, 99.9%) were heated in a flowing nitrogen atmosphere (Nippon Gases, 99.999%, purified with an oxysorb cartridge) utilising a custom water-cooled radio frequency furnace^61^. After the complete reaction of the samples, they were crushed and sieved through a 100 mesh. The preparation of samples was carried out in a protective argon atmosphere.

### Powder X-ray diffraction

PXRD data of finely ground *Ln*N powders was collected on a Huber G670 imaging plate Guinier camera (2*Θ*_max_ = 100°) using a curved germanium (111) monochromator and Cu-K_α1_ (*Ln* = La, Ce, Sm, Er, Yb) or Co-K_α1_ radiation (*Ln* = Eu) at 293(1) K. Co-K_α1_ radiation was used for EuN to avoid fluorescence effects that are caused by the Cu-K_α1_ energy alike L_1_-edge of Eu, hence improving the signal to noise ratio of the diffraction data^62,63^. The powder samples were placed between Kapton foils to avoid oxidation in air. PXRD data of the high-temperature oxidised *Ln*N (*Ln* = La, Ce, Sm, Eu, Er, Yb) compounds in this investigation were collected on a Bruker D8 diffractometer equipped with a Lynxeye XE-T detector in Bragg–Brentano configuration using Cu-K_α1,2_ radiation (*λ* = 1.54184 Å) at room temperature. The powder samples were placed on a thin Vaseline layer on Si-zero background holders closed by an airtight Kapton dome. Measurements were performed in the range of 20° ≤ 2*θ* ≤ 85° with a step size of (2*θ*) = 0.02° and a counting time of 2 s per step. Data analysis was performed via the Rietveld method as implemented in the program GSAS-II^64^. The PXRD characterisation of pristine and high-temperature oxidised *Ln*N powders (*Ln* = Pr, Nd, Gd, Tb, Dy, Ho, Tm, Lu) was reported previously^1^.

### Thermogravimetric analysis

The thermogravimetric characterisation of the high-temperature oxidation behaviour of *Ln*N (*Ln* = La, Ce, Sm, Eu, Er, Yb) powders with a NETZSCH STA 449C in thermogravimetric and differential scanning calorimetry setup (TGA-DSC) equipped with a thermocouple type S (σ_T_ = ± 1 °C) has been adapted from previous works^1^. Powder samples of 20 mg were heated in Pt/Rh crucibles (NETZSCH) to 900 °C under flowing synthetic air at 10 °C/min rate. The oxidation residues were analysed by PXRD.

### High temperature oxide melt drop solution calorimetry

High temperature calorimetry experiments were performed using a Calvet Calorimeter (Setaram AlexSys 1000)^34,65^, where each of the two calorimeter chambers was maintained at 973 K and contained a quartz crucible with 30 g of molten sodium molybdate solvent (3 NaO · 4 MO_3_). The sample chambers were kept under flowing oxygen gas (A-L Compressed Gases, 99.5%) with ~60 mL/min, which was also bubbled through molten salt solvents at 4 mL/min to maintain oxidising conditions. Prior to sample measurements, the calorimeter was calibrated using Al_2_O_3_ calibrants, which were dropped into empty quartz crucibles in separate experimental runs. Pellets of around 5 mg *Ln*N (*Ln* = La, Ce, Pr, Nd, Sm, Eu, Gd, Tb, Dy, Ho, Er, Tm, Yb, Lu) with a diameter of 1 mm were prepared before each respective experiment and sealed in plastic bags under a protective nitrogen atmosphere. In order to minimise air contact of the sample pellets, the bags were cut open right before the drop into the calorimeter, where they dissolved in the molten salt solvent. During the initial drop experiments of CeN, visible light flashes were observed, indicating ignition before dissolution. To prevent unintended oxidation, CeN samples were encapsulated in Rutile-structured TiO_2_ pellets (1.5 mm diameter), isolating them from air contact until dissolution. PXRD structure analysis of TiO_2_ is provided in Supplementary Information Note 1, Fig. S1 with Rietveld analysis results summarised in Table S1. The standard enthalpy of formation was calculated by two constructed thermocycles which were based on the observed high-temperature oxidation behaviour for the respective *Ln*N compounds described in this investigation and elsewhere^1^.

## Supplementary information

Transparent Peer Review file

Supplementary Information File

## Data Availability

The data that support the findings of this study is available in an open access Zenodo repository, https://doi.org/10.5281/zenodo.22296171.
