# Supplementary material for: The energetics of high temperature oxidation and formation mechanisms in binary lanthanide nitrides (LnN)
Source: Commun Chem. 2026 Oct 7;9:317. doi: 10.1038/s42004-026-02236-8 (PMC13645747; doi:10.1038/s42004-026-02236-8)
Supplement: Supplementary file 1 [file 42004_2026_2236_MOESM1_ESM.pdf]

nature portfolio

Peer Review File

The Energetics of High Temperature Oxidation and Formation Mechanisms in Binary Lanthanide Nitrides (LnN)

Corresponding Author: Dr Gabriel Murphy

This file contains all reviewer reports in order by version, followed by all author rebuttals in order by version.

Attachments originally included by the reviewers as part of their assessment can be found at the end of this file.

Version 0:

Reviewer comments:

Reviewer #1

(Remarks to the Author)
The paper supplied for review presents the experimental synthesis of lanthanide nitride materials and the determination of their thermochemical parameters using calorimetry. The aim of the paper is to quantify the observation that the incorporation of lanthanide nitrides into uranium nitride fuels, either deliberately or through fission lowers the combustion risk of the fuel if exposed to oxygen or water. In a general sense this manuscript and the associated data are expertly presented and collected. The analysis is clear and straightforward. Some issues to address are delineated below and should be addressed and/or investigated prior to publication.

1. In this reviewer's opinion the "tetrad effect" is overstated and very poorly referenced or discussed. The tetrad effect is a nuance observed in some studies, principally separations chemistry, whose origin is debatable. The attribution of the observed data in this study to the tetrad effect is not at all clear. At first pass, one would expect some systematic change in the thermochemical values across the lanthanide series with discontinuities in that trend arising around the half-filled shell at Gd and the end member Lu, with Yb, Tb, and Eu showing anomalous behavior. The data acquired here are difficult to discern any periodic trend at all, let alone the nuance of the tetrad effect. This is further complicated by the fact that the measured values in this paper do not agree with those previously measured, referenced and presented in Figure S2. Why is this? The authors state that their data are in agreement but there is a clear discrepancy between the trans-gadolinium data and the cis-gadolinium data. The literature values referenced show a trend in thermochemistry that is more expected based on atomic properties than the data being presented in the current study. This needs to be addressed more directly and discussed.

2. Along the lines of point 1 above, the lanthanide starting materials were procured as metals and the purity from the supplier is reported. However, I would require that independent analysis of these metals be conducted to verify their purity. The data from the light rare earths is consistent with earlier literature studies but the heavy rare earths are scattered and systematically lower than those previously reported. Is this an issue of material purity? These impurities would not be obvious in the powder diffraction and should be investigated by elemental analysis. Certainly the data measured here show a clear thermochemical reasoning for the stabilization of UN upon incorporation of LnN materials but I am concerned that there is a systematic flaw in the experiment that can be easily addressed by some analysis of the starting materials.

Reviewer #2

(Remarks to the Author)
Review attached

Version 1:

Reviewer comments:

Reviewer #1

(Remarks to the Author)
The author's have responded thoughtfully and adequately to my concerns. I recommend that this be published.

Reviewer #2

(Remarks to the Author)
I appreciate the authors' response and recommend publication.

Open Access This Peer Review File is licensed under a Creative Commons Attribution 4.0 International License, which permits use, sharing, adaptation, distribution and reproduction in any medium or format, as long as you give appropriate credit to the original author(s) and the source, provide a link to the Creative Commons license, and indicate if changes were made.
In cases where reviewers are anonymous, credit should be given to 'Anonymous Referee' and the source.
The images or other third party material in this Peer Review File are included in the article's Creative Commons license, unless indicated otherwise in a credit line to the material. If material is not included in the article's Creative Commons license and your intended use is not permitted by statutory regulation or exceeds the permitted use, you will need to obtain permission directly from the copyright holder.
To view a copy of this license, visit https://creativecommons.org/licenses/by/4.0/

Reviewer #1 (Remarks to the Author):

The paper supplied for review presents the experimental synthesis of lanthanide nitride materials and the determination of their thermochemical parameters using calorimetry. The aim of the paper is to quantify the observation that the incorporation of lanthanide nitrides into uranium nitride fuels, either deliberately or through fission lowers the combustion risk of the fuel if exposed to oxygen or water. In a general sense this manuscript and the associated data are expertly presented and collected. The analysis is clear and straightforward. Some issues to address are delineated below and should be addressed and/or investigated prior to publication.

1. In this reviewer's opinion the "tetrad effect" is overstated and very poorly referenced or discussed. The tetrad effect is a nuance observed in some studies, principally separations chemistry, whose origin is debatable. The attribution of the observed data in this study to the tetrad effect is not at all clear. At first pass, one would expect some systematic change in the thermochemical values across the lanthanide series with discontinuities in that trend arising around the half-filled shell at Gd and the end member Lu, with Yb, Tb, and Eu showing anomalous behavior. The data acquired here are difficult to discern any periodic trend at all, let alone the nuance of the tetrad effect. This is further complicated by the fact that the measured values in this paper do not agree with those previously measured, referenced and presented in Figure S2. Why is this? The authors state that their data are in agreement but there is a clear discrepancy between the trans-gadolinium data and the cis-gadolinium data. The literature values referenced show a trend in thermochemistry that is more expected based on atomic properties than the data being presented in the current study. This needs to be addressed more directly and discussed.

Response

We thank the Reviewer for the critical but very key comments regarding firstly the claim of lanthanide tetrad effect, and secondly the discrepancies with previous literature data, which are separately addressed:

Tetrad effect: We agree with the Reviewer that speculating on the lanthanide tetrad effect is not appropriate without further deeper chemical insight/investigation that is beyond the scope of the study. We have accordingly removed all references to the lanthanide tetrad effect throughout the manuscript. When considering the trends in our data, particularly the significant drop in formation enthalpy when DyN is encountered in the series trans-Gd, this does appear consistent with half shell electron filling trends. Increasing to be above a half-filled f-electron levels, since Dy^{3+} is $4f^7$ and pairing would start to occur. We have included small discussion on this in the manuscript. But we emphasize that this is only small speculation and not a determined conclusion or derivation from the work.

Regarding the specific discrepancy with the literature data, this is also a pertinent point. Firstly, it needs to be emphasized that the formation enthalpies have been determined using very different techniques between the use of high temperature calorimetry in this study compared to the KEMS of the literature study. The KEMS measurements derive formation enthalpies indirectly from high-temperature vapour equilibrium measurements and therefore relies on several assumptions and auxiliary thermodynamic quantities. In contrast, high-temperature oxide melt calorimetry directly measures the enthalpy of the chemical reaction, from which the standard formation enthalpy is obtained through a well-defined thermochemical cycle. As a direct calorimetric method, it inherently reflects the energetics of the real material, including effects arising from slight non-stoichiometry or electronic stabilisation, without relying on an equilibrium model. It can be argued there is some commonality in the formation enthalpies for the pre-Gd Ln's comparing the data but also strong differences are also seen in this region: For instance, we report considerably lower formation enthalpy values that are often out of the uncertainty measurement range for the corresponding KEMS measurements, for instance Gd, Sm, Nd and also very noticeably Eu which has a difference of approximately -120 kJ mol^{-1} which thermodynamically is a considerable difference. Moreover, it appears there is little agreement between our data and that of the previous KEMS measurements. Pertinently, considering the inherent and strong differences in the experimental measurement and the assumptions involved in KEMS measurements, we argue it is not appropriate to bench mark our results against there's. Instead, we offer thermodynamic data based on high temperature calorimetry using well defined thermodynamic cycles.

2. Along the lines of point 1 above, the lanthanide starting materials were procured as metals and the purity from the supplier is reported. However, I would require that independent analysis of these metals be conducted to verify their purity. The data from the light rare earths is consistent with earlier literature studies but the heavy rare earths are scattered and systematically lower than those previously reported. Is this an issue of material purity? These impurities would not be obvious in the powder diffraction and should be investigated by elemental analysis. Certainly, the data measured here show a clear thermochemical reasoning for the stabilization of UN upon incorporation of *Ln*N materials but I am concerned that there is a systematic flaw in the experiment that can be easily addressed by some analysis of the starting materials.

Response

The Reviewer raises a good question regarding material purity and whether it can affect the determined formation enthalpies, since heavy rare earths (HREE) can be affected by contamination of other HREEs. Unfortunately, most of the Ln metals were consumed as a part of the nitride synthesis work preventing their direct analysis. As nitrides, all *Ln*N samples were precisely characterized by powder X-ray diffraction combined with Rietveld analysis, and detectable impurity phases were identified and reported when present, including minor impurities for instance Ta in LuN. We showed that the maximum amount of impurity present from XRD analysis to be 1.9 wt % (Uhlemann et al. NPJ Mat. Degrad. 2026, 10.1038/s41529-026-00827-3). In considering previous thermochemical studies by Buyukkilic et al. (Buyukkilic, S., et al. Solid-State Ionics 2012, 10.1016/j.ssi.2012.08.017) who examined Nd and Sm doping of CeO_2 thermodynamically up to 30 mol %, a maximum change in enthalpy of only a few kJ mol^{-1} was observed. Furthermore and more critically, in considering the enthalpy results of the present study of the heavier *Ln*Ns which differ by approximately 50 kJ/mol maximum value between different *Ln*N samples, if a similar impurity were to occur across these, caused by metal impurities in HREE processing at levels below XRD detection limits or up to 1.9 wt% as found previously, the variation in value is expected to fall well within the uncertainty limits of the calorimetric

measurement. Consequently, even if such impurities were present, they cannot reasonably account for the approximately 200 kJ mol^{-1} break observed between the pre- and trans-gadolinium formation enthalpies. We therefore consider trace lanthanide impurities unlikely to be the origin of the observed break and any trace amounts present to only minimally effect the determined formation enthalpies that are accounted for by the uncertainty of the measurement. Moreover, considering the robustness and precision of the calorimetry method, we argue the results are a true reflection of the thermodynamic properties of the lanthanide nitrides and differences to the previous KEMS are more linked to associated limitations in measurement and assumptions required for that measurement type.

Reviewer #2 (Remarks to the Author):

Uhlemann, Guo, Murphy, and coworkers report a systematic study on the energetics of the oxidation of LnN series both by oxygen and water excluding radioactive promethium. The results are compared to previously published UN. The data was collected by temperature oxide melt calorimetry with the melt being a sodium-tungsten oxide mixture. Powder X-ray diffraction was used to determine the structure type after LnN ignition. The results show that the LnN materials were more thermodynamically favored than UN where it is hypothesized that the LnN formed from the fission of UN will stabilize the material to oxidation. The manuscript is well written, and I was surprised that a material as important as LnN has not been investigated thermodynamically in this amount of detail. I recommend publication Communications Chemistry after a few minor revisions below.

Line 49: change spectrometric to spectrometry

Response:

We thank the Reviewer for highlighting this mistake and corrected the manuscript accordingly.

Line 90: consider explaining why cobalt radiation was used for only Eu

Response:

We thank the reviewer for this suggestion and amended the manuscript accordingly, particularly that Co avoids fluorescence issues associated with Cu based radiation on Eu.

Line 94: change CuKα1,2 to Cu-$K_{\alpha1,2}$ for consistency

Response:

We thank the Reviewer for highlighting this improvement and adjusted the manuscript accordingly.

Line 144: change *P*63/*m* to *P*6_3/*m*

Response:

We thank the Reviewer for marking this mistake and corrected the manuscript accordingly.

Line 146: change *Ln*$^{+3}$N to *Ln*$^{3+}$N

Response:

We thank the Reviewer for highlighting the mistake and corrected the manuscript accordingly.

Line 158: change *P*63/*m* to *P*6_3/*m*

Response:

We thank the Reviewer for highlighting the mistake and corrected the manuscript accordingly.

Table 2: fix the space groups to their standard format for 194 and 12 (*P*6_3/*m* and *C*2/*m*)

Response:

We thank the Reviewer for highlighting the mistakes and corrected the manuscript accordingly.

Line 227: There seems to be a new paragraph on accident

Response:

We thank the Reviewer for highlighting this error and corrected the autoformatting of the reference to the table.

Table 5: Is there any possibility that Pr6O11 or Tb4O7 is ever formed? I know these oxides are the most common under ambient conditions.

Response:

We thank the Reviewer for the question. Yes, both oxides $PrO_{1.833}$ and $TbO_{1.75}$ were observed in the 900 °C TGA-DSC oxidation runs with air upon heating the respective nitride to T > 500 °C.[1] However, for the High-temperature oxide melt calorimetry their formation is very unlikely, due to highly oxidizing conditions owed to the free ionic oxide species in the molten sodium molybdate salt solvent.

Figure 5: Consider clarifying or restating that the tetravalent ionic radii are used for some instances

for Ce, Pr, Tb, and U

Response:

We thank the Reviewer for the suggestion and agree that the manuscript could be improved for better clarity in this matter. We amended the manuscript accordingly with improved descriptions of the ionic radii used for plotting the Gibbs energies.

Table 7: consider adding the reference for the UN

Response:

We thank the Reviewer for this suggestion. We agree that the empty cell in Table 7 can cause confusion and amended the manuscript accordingly.

I appreciate the editor and the authors for their time and congratulate the authors on their results.

Response: We thank the Reviewer for the helpful and very constructive Review.

References:

1 Uhlemann, P. *et al.* The Ambient- and High-Temperature Oxidation Behaviour of $U^{3+}N$ and $Ln^{3+}N$ Compounds Relevant to Spent Nuclear Fuel. (2026). https://doi.org:https://doi.org/10.1038/s41529-026-00827-3

2 Buyukkilic, S., Shvareva, T. & Navrotsky, A. Enthalpies of formation and insights into defect association in ceria singly and doubly doped with neodymia and samaria. *Solid State Ionics* **227**, 17-22 (2012). https://doi.org:https://doi.org/10.1016/j.ssi.2012.08.017

Review for COMMSCHEM-26-0465

Uhlemann, Guo, Murphy, and coworkers report a systematic study on the energetics of the oxidation of LnN series both by oxygen and water excluding radioactive promethium. The results are compared to previously published UN. The data was collected by temperature oxide melt calorimetry with the melt being a sodium-tungsten oxide mixture. Powder X-ray diffraction was used to determine the structure type after LnN ignition. The results show that the LnN materials were more thermodynamically favored than UN where it is hypothesized that the LnN formed from the fission of UN will stabilize the material to oxidation. The manuscript is well written, and I was surprised that a material as important as LnN has not been investigated thermodynamically in this amount of detail. I recommend publication *Communications Chemistry* after a few minor revisions below.

Line 49: change spectrometric to spectrometry

Line 90: consider explaining why cobalt radiation was used for only Eu

Line 94: change $CuK\alpha_{1,2}$ to $Cu\text{-}K_{\alpha 1,2}$ for consistency

Line 144: change $P63/m$ to $P6_3/m$

Line 146: change $Ln^{+3}N$ to $Ln^{3+}N$

Line 158: change $P63/m$ to $P6_3/m$

Table 2: fix the space groups to their standard format for 194 and 12 ($P6_3/m$ and $C2/m$)

Line 227: There seems to be a new paragraph on accident

Table 5: Is there any possibility that Pr_6O_{11} or Tb_4O_7 is ever formed? I know these oxides are the most common under ambient conditions.

Figure 5: Consider clarifying or restating that the tetravalent ionic radii are used for some instances for Ce, Pr, Tb, and U

Table 7: consider adding the reference for the UN

I appreciate the editor and the authors for their time and congratulate the authors on their results.
