# Supplementary material for: The energetics of high temperature oxidation and formation mechanisms in binary lanthanide nitrides (LnN)
Source: Commun Chem. 2026 Oct 7;9:317. doi: 10.1038/s42004-026-02236-8 (PMC13645747; doi:10.1038/s42004-026-02236-8)
Supplement: Supplementary file 2 [file 42004_2026_2236_MOESM2_ESM.pdf]

Supplementary Information

The Energetics of High Temperature Oxidation and Formation Mechanisms in Binary Lanthanide Nitrides (*Ln*N)

Pascal Uhlemann,[1]* W. Vance,[2] M. P. Heaney,[2] N. Yaw,[2] C. Schreinemachers,[1] P. Kegler,[1] P. Höhn,[3] X. Guo,[2]* G. Modolo,[1] and G. L. Murphy[1]*

[1]*Institute of Fusion Energy & Nuclear Waste Management (IFN-2), Forschungszentrum Jülich GmbH, 52428, Jülich, Germany*

[2]*Alexandra Navrotsky Institute for Experimental Thermodynamics, Department of Chemistry, Washington State University, Pullman, Washington 99164, United States*

[3]*Max-Planck-Institute for Chemical Physics of Solids, 01187 Dresden, Germany*

*corresponding authors: Pascal Uhlemann (p.uhlemann@fz-juelich.de), X. Guo (x.guo@wsu.edu) and G. L. Murphy (g.murphy@fz-juelich.de)

Supplementary Information Note 1 – Rietveld refinement of TiO_2 Rutile structure

Supplementary Figure S1 show the Rietveld refinement of TiO_2 in rutile structure used to protect the CeN for high-temperature oxide melt calorimetry experiments. The results determined from Rietveld analysis as performed in the refinement program GSAS-II[1] are summarised in Table S1. The PXRD of finely ground TiO_2 powder was collected on a PANalytical X'Pert PRO diffractometer in Bragg-Brentano configuration equipped with an 1D- X'Celerator detector using $CuK_{\alpha1,2}$ radiation. Measurements were performed in the range of $10° \leq 2\theta \leq 100°$ with a step size of $(2\theta) = 0.001°$.

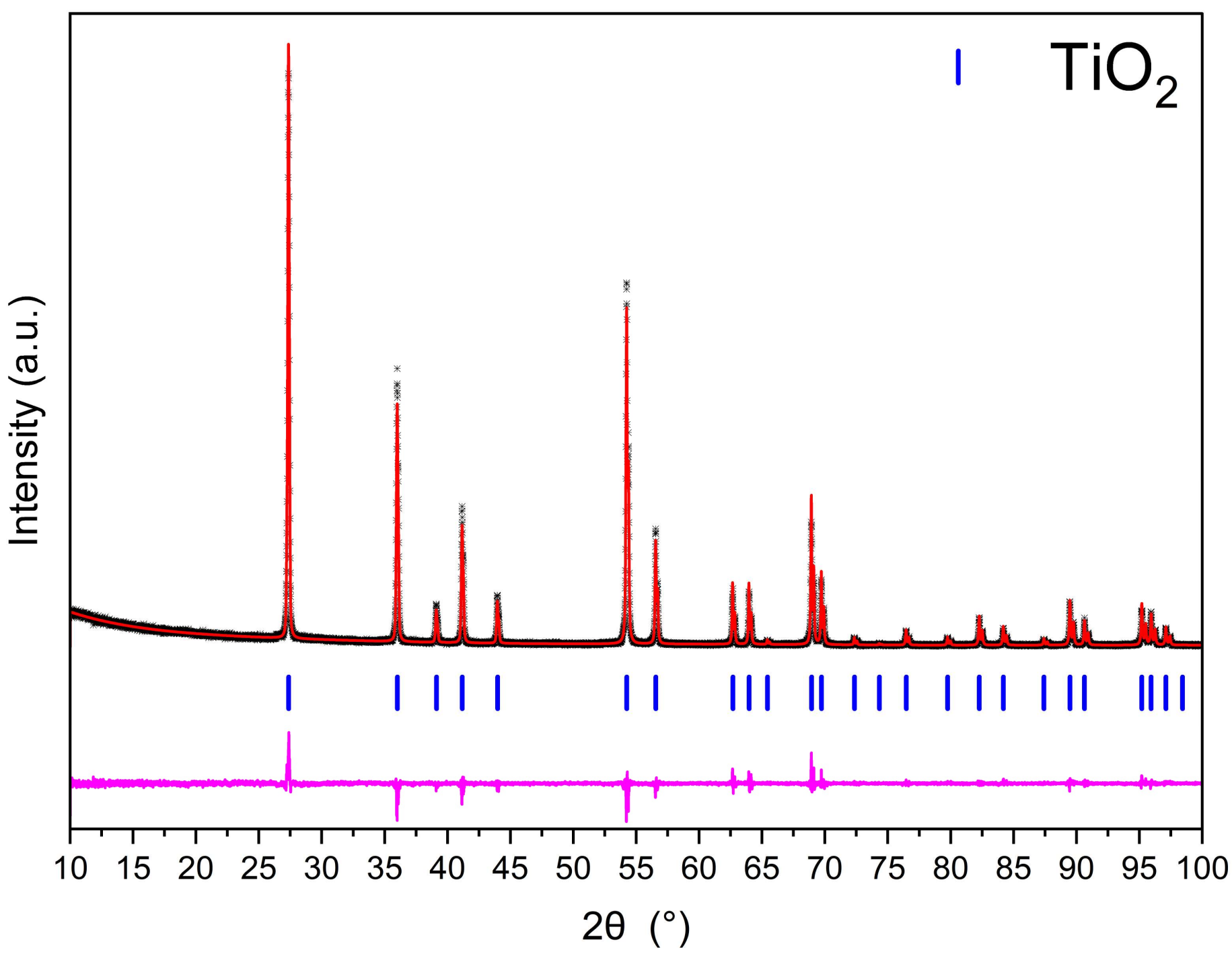

Figure S1. Rietveld profile of TiO_2 sample in Rutile structure used to protect CeN from oxidation upfront dissolution in high temperature oxide melt calorimetry. The black crosses, red and pink lines and vertical blue respectively represent observed data, calculated profile, difference profile and allowed reflections of TiO_2 crystallising in tetragonal *P*42/*mnm* structure.

Table S1. Refined lattice parameters determined from Rietveld refinements against PXRD data, associated fitting factors and literature lattice parameters for TiO_2 in Rutile structure. Additional refinement details are shown in Supplementary Information Note 2, Table S2. Note uncertainties are reported directly from the Rietveld analysis with no correction.

Compound	Structure and Space Group	Lattice Parameters (Å)	Fitting factors (%)	Literature lattice parameter (Å)
TiO_2	*P*42/*mnm* (136)	*a* = 4.593310(18)<br>*c* = 2.958805(16)	R = 8.33<br>R_w = 11.07	*a* = 4.59245(4)<br>*c* = 2.95732(3)<br>2

Supplementary Information Note 2 – Rietveld refinement parameters

Supplementary Table S2 summarises additional refinement details performed using Rietveld analysis with the program GSAS-II[1] against the main compounds determined by PXRD measurements used in this work.

Table S2. Summary of additional refinement results determined from Rietveld analysis using the program GSAS-II.

Phase	$R_{w\text{-}bkg}$ (%)	$R_{w,min}$ (%)	GOF
Pristine Nitride Phases, see Table 1			
LaN	3.47	3.95	0.88
CeN	1.60	1.37	1.17
SmN	2.81	3.20	0.88
EuN	4.84	1.70	2.84
ErN	3.83	1.40	2.73
YbN	3.95	1.39	2.85
High Temperature Oxidised Phases, see Table S2			
La_2O_3	2.57	1.61	1.60
CeO_2	3.31	1.61	2.06
Sm_2O_3	2.02	1.63	1.24
Eu_2O_3	2.08	1.63	1.28
Er_2O_3	2.62	1.32	1.99
Yb_2O_3	3.19	1.35	2.37
TiO_2 in Rutile structure			
TiO_2	11.07	9.07	1.22

Supplementary Information Note 3 – Enthalpies

Supplementary Table S3 summarises additional enthalpies determined from high temperature oxide melt calorimetry measurements, as well as literature data which was used to calculate the formation enthalpies of the *Ln*N compounds. Figure S2 compares calculated formation enthalpies of the *Ln*N compounds that were measured against literature date determined via Knudsen Cell effusion mass spectrometry (KEMS).[3]

Table S3: Summary of enthalpies from literature and experimentally determined to calculate $\Delta H_f^{\circ}(LnN)$. Formation enthalpies for lanthanide oxides ΔH_f° (Ln_2O_3/LnO_2) and dissolution enthalpies of lanthanide oxides in sodium molybdate at 700°C ΔH_2 were received from literature. Dissolution enthalpies for *Ln*N ΔH_{ds} (*Ln*N) and oxidation enthalpies for *Ln*N at 700°C in sodium molybdate ΔH_{ox} (*Ln*N) with their associated errors were experimentally determined in this investigation. All values are summarized in kJ mol^{-1}.

Ln	ΔH_f° (Ln_2O_3/LnO_2)	ΔH_2	ΔH_{ds} (*Ln*N)	$2\sigma(\Delta H_{ds})$	ΔH_{ox} (*Ln*N)	$\sigma(\Delta H_{ox})$	Ref
La	-1791.6 ± 2.0	-190.70 ± 2.46	-696.52	12.92	-578.88	13.0	4,5
Ce	-1090.4 ± 0.8	73.39 ± 1.65	-640.08	7.92	-702.05	8.1	4,5
Pr	-954.2 ± 5.0	-29.71 ± 1.35	-674.08	15.31	-632.96	15.4	6,7
Nd	-1813.1 ± 0.8	-163.36 ± 3.44	-635.30	8.82	-531.33	9.0	5,8
Sm	-1823.0 ± 4.0 (m)	-153.62 ± 2.86	-624.61	11.02	-525.52	11.1	4,5
Eu	-1662.5 ± 6.0 (c)	-129.24 ± 2.12	-589.87	6.49	-502.97	6.6	4,5
Gd	-1854.0 ± 15.0 (c)	-148.54 ± 1.60	-655.27	16.17	-558.72	16.2	5,6
Tb	-972.1 ± 5.0	-28.132 (t)	-664.36	10.37	-624.82	10.4	4,9
Dy	-1863.4 ± 5.0	-114.88 ± 2.22	-522.17	12.38	-442.44	12.4	4,5
Ho	-1883.3 ± 8.2	-111.72 ± 3.68	-502.05	3.98	-423.90	4.4	4,5
Er	-1900.1 ± 6.5	-105.26 ± 2.48	-457.99	15.96	-383.07	16.0	4,5
Tm	-1889.3 ± 5.7	-97.12 ± 2.38	-525.67	10.89	-454.83	11.0	4,5
Yb	-1814.5 ± 6.0	-98.46 ± 3.18	-449.68	6.77	-378.17	7.0	4,5
Lu	-1877.0 ± 7.7	-96.90 ± 1.90	-459.52	0.70	-388.79	1.2	4,5

(c) cubic structure, (m) monoclinic structure, (t) theoretical calculated value

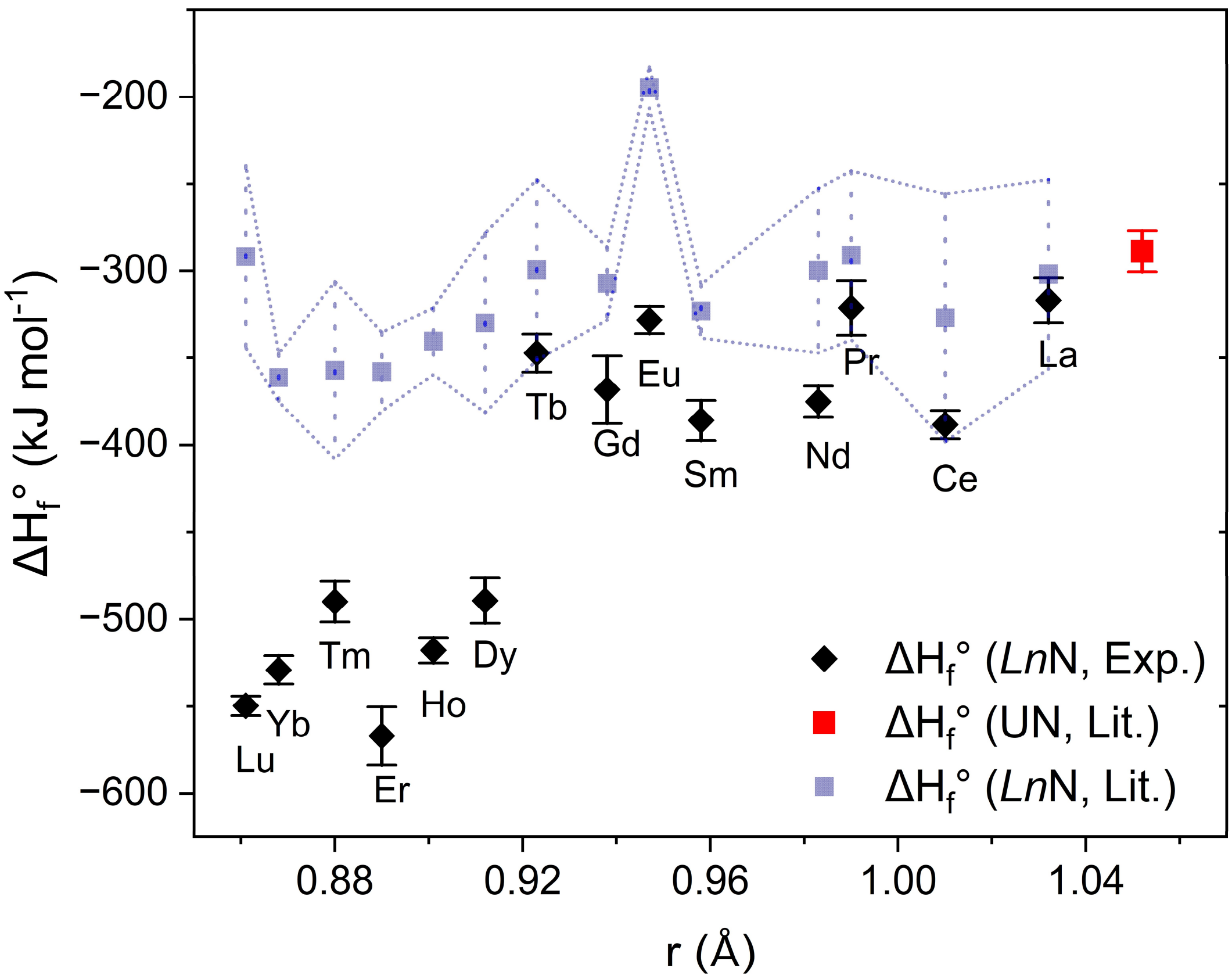

Figure S2. Experimentally determined ΔH_f° for *Ln*N compounds where *Ln* = La, Ce, Pr, Nd, Sm, Eu, Gd, Tb, Dy, Ho, Er, Tm, Yb and Lu plotted against the ionic radii r of the Ln^{3+} cation in 6-fold coordination and comparison against literature formation enthalpies ΔH_f° based on KEMS measurements.[3] The literature value for the ΔH_f° of UN was determined by the same methodology is plotted for highlighting the trend of ΔH_f° against r.[10]

Supplementary Information Note 4 – Gibbs energies

Supplementary Table S4 summarises additional enthalpies and entropies from literature data which were used to calculate the Gibbs energies of formation and oxidation of *Ln*N and UN. The literature data of entropies for *Ln*N is scarce and data was solely derived for LaN and CeN, which was applied for calculation of the thermodynamic data of all the other *Ln*N. Supplementary Table S5 summarises reaction enthalpies and entropies calculated prior to the Gibbs energies of formation and oxidation.

Table S4: Summary of literature entropy data for *Ln* metals, *Ln* oxides, *Ln* hydroxides and *Ln*N, in addition to the formation enthalpy and entropies of UN, UO_2, H_2O, NH_3 and H_2 used to calculate ΔG_f°, ΔG_{ox}° and ΔG_{hyd}° at 298.15 K.

Compound	ΔH_f° (kJ mol^{-1})	S°(J mol^{-1} K^{-1})	Ref
LaN		44.350	9
La_2O_3		127.32 ± 0.84	11
$La(OH)_3$	-1416.1	117.81	12
La		56.902	9
CeN		44.350	9
CeO_2		62.29 ± 0.07	13
$Ce(OH)_3$	-1416.1	129.40	12
Ce		69.454	9
PrO_2		80.80 ± 2.00	14
Pr		73.931	9
$Pr(OH)_3$	-1413.8	131.70	12
Nd_2O_3		158.70 ± 1.00	11
$Nd(OH)_3$	-1415.6	129.87	12
Nd		71.086	9
$Sm_2O_3^{(m)}$		150.60 ± 0.30	15
$Sm(OH)_3$	-1406.6	125.80	12
Sm		69.496	9
$Eu_2O_3^{(m)}$		143.50 ± 0.50	16
$Eu(OH)_3$	-1319.1	119.88	12
Eu		77.822	9
$Gd_2O_3^{(m)}$		157.10 ± 0.20	17
$Gd(OH)_3$	-1408.9	126.63	12
Gd		67.948	9
TbO_2		86.90 ± 3.00	14
$Tb(OH)_3$	-1414.8	128.37	12
Tb		73.304	9
Dy_2O_3		149.80 ± 0.15	18
$Dy(OH)_3$	-1428.4	130.30	12
Dy		74.894	9
Ho_2O_3		156.38 ± 0.15	18
$Ho(OH)_3$	-1431.1	130.04	12
Ho		75.019	9
Er_2O_3		153.13 ± 0.15	18
$Er(OH)_3$	-1432.5	128.60	12
Er		73.178	9
Tm_2O_3		139.70 ± 0.40	19

$Tm(OH)_3$	-1421.1	126.50	12
Tm		74.015	9
Yb_2O_3		133.10 ± 0.30	15
$Yb(OH)_3$	-1395.5	118.60	12
Yb		59.831	9
Lu_2O_3		109.96 ± 0.13	19
$Lu(OH)_3$	-1427.0		20
Lu		50.961	9
UN	-288.8 ± 5.9	62.22	10,21
UO_2	-1084.9 ± 1.0	77.03 ± 0.20	22,23
U		50.292	9
$H_2O_{(g)}$	-241.8	192.77 ± 0.05	23
NH_3	-45.9	188.84 ± 0.01	23
H_2		130.68 ± 0.003	23

m = monoclinic, g = gaseous

Table S5: Summary of calculated reaction formation entropies $\Delta S_{rct,f}^{\circ}$, reaction oxidation enthalpies $\Delta H_{rct,ox}^{\circ}$ and entropies $\Delta S_{rct,ox}^{\circ}$, and reaction hydrolysis $\Delta H_{rct,hdy}^{\circ}$ and entropies $\Delta S_{rct,hyd}^{\circ}$, used to calculate ΔG_f°, ΔG_{ox}° and ΔG_{hyd}° at 298 K.

Element	$\Delta S_{rct,f}^{\circ}$ (J mol^{-1} K^{-1})	$\Delta H_{rct,ox}^{\circ}$ (kJ mol^{-1})	$\Delta S_{rct,ox}^{\circ}$ (J mol^{-1} K^{-1})	$\Delta H_{rct,hyd}^{\circ}$ (kJ mol^{-1})	$\Delta S_{rct,hyd}^{\circ}$ (J mol^{-1} K^{-1})
La	-43.66	-584.86	-38.75	-300.28	-330.12
Ce	-56.21	-713.47	-91.41	-288.69	-262.14
Pr	-60.69	-644.37	-72.90	-286.39	-327.63
Nd	-57.84	-537.31	-23.06	-288.22	-274.91
Sm	-56.25	-531.50	-27.11	-292.29	-253.94
Eu	-64.58	-502.90	-30.66	-298.21	-222.37
Gd	-54.71	-547.55	-23.86	-291.46	-274.18
Tb	-60.06	-625.36	-66.80	-289.72	-301.60
Dy	-61.65	-448.42	-27.51	-287.79	-173.80
Ho	-61.78	-429.88	-24.22	-288.05	-147.93
Er	-59.94	-389.05	-25.84	-289.49	-99.67
Tm	-60.77	-460.81	-32.56	-291.59	-164.80
Yb	-46.59	-384.15	-35.86	-299.49	-97.59
Lu	-37.72	-394.77	-47.43	-291.59	-110.81
U	-19.18	-807.53	-94.53	-104.75	-327.17

References

1 Toby, B. H. & Dreele, R. B. v. GSAS-II: the genesis of a modern open-source all purpose crystallography software package. *Journal of Applied Crystallography* **46**, 544–549 (2013). https://doi.org:10.1107/s0021889813003531

2 Huang, H. *et al.* Structural, Magnetic, and Electronic Properties of the $Cr_{1-x}Ti_xO_2$ Solid. *Inorganic Chemistry* **61**, 1391–1400 (2022). https://doi.org:10.1021/acs.inorgchem.1c02923

3 Kordis, J. & Gingerich, K. A. Heats of vaporization and standard heats of formation of rare earth mononitrides. *Journal of Nuclear Materials* **66**, 197–199 (1977). https://doi.org:10.1016/0022-3115(77)90147-7

4 Cordfunke, E. H. P. & Konings, R. J. M. The enthalpies of formation of lanthanide compounds. *Thermochimica Acta* **375**, 65–79 (2001). https://doi.org:10.1016/s0040-6031(01)00510-x

5 Ushakov, S. V., Helean, K. B., Navrotsky, A. & Boatner, L. A. Thermochemistry of rare-earth orthophosphates. *Journal of Materials Research* **16**, 2623–2633 (2001). https://doi.org:10.1557/jmr.2001.0361

6 Morss, L. R. & Konings, R. J. M. in *Binary Rare Earth Oxides* (eds G. Adachi, N. Imanaka, & Z. C. Kang) 163–188 (Springer Netherlands, 2004).

7 Ushakov, S. V., Cheng, J., Navrotsky, A., Wu, J. R. & Haile, S. M. Formation Enthalpies of Tetravalent Lanthanide Perovskites by High Temperature Oxide Melt Solution Calorimetry. *MRS Proceedings* **718** (2002). https://doi.org:10.1557/proc-718-d7.17

8 Huntelaar, M. E. *et al.* The thermodynamic properties of $Ce_2O_{3(s)}$ fromT→ 0 K to 1500 K. *The Journal of Chemical Thermodynamics* **32**, 465–482 (2000). https://doi.org:10.1006/jcht.1999.0614

9 Barin, I. *Thermochemical Data of Pure Substances*. (Wiley, 1995).

10 Goncharov, V. G. *et al.* Energetics of oxidation and formation of uranium mononitride. *Journal of Nuclear Materials* **569**, 153904 (2022). https://doi.org:10.1016/j.jnucmat.2022.153904

11 Justice, B. H. & Westrum, E. F. Thermophysical properties of the lanthanide oxides. I. Heat capacities, thermodynamic properties, and some energy levels of Lanthanum(III) and Neodymium(III) oxides from 5 to 350K. *The Journal of Physical Chemistry* **67**, 339–345 (1963). https://doi.org:10.1021/j100796a031

12 Diakonov, I. I., Ragnarsdottir, K. V. & Tagirov, B. R. Standard thermodynamic properties and heat capacity equations of rare earth hydroxides. *Chemical Geology* **151**, 327–347 (1998). https://doi.org:10.1016/s0009-2541(98)00088-6

13 Westrum, E. F. & Beale, A. F. Heat capacities and Chemical Thermodynamics of Cerium(III) Flouride and of Cerium(IV) Oxide from 5 to 300°K. *The Journal of Physical Chemistry* **65**, 353–355 (1961). https://doi.org:10.1021/j100820a041

14 Konings, R. J. M. *et al.* The Thermodynamic Properties of the f -Elements and their Compounds. Part 2. The Lanthanide and Actinide Oxides. *Journal of Physical and Chemical Reference Data* **43** (2014). https://doi.org:10.1063/1.4825256

15 Justice, B. H. & Westrum, E. F. Thermophysical properties of the lanthanide oxides. II. Heat capacities, thermodynamic properties, and some energy levels of Samarium(III),

Gadolinium(III), and Ytterbium(III) oxides from 10 to 350°K. *The Journal of Physical Chemistry* **67**, 345–351 (1963). https://doi.org:10.1021/j100796a032

16 Lyutsareva, N. S., Berezovskii, G. A. & Paukov, I. E. Heat Capacity of Two Modifications of Eu_2O_3 with a Range of 8–300 K. *Zh. Fiz. Khim* **68**, 1179–1182 (1994).

17 Konings, R. J. M., van Miltenburg, J. C. & van Genderen, A. C. G. Heat capacity and entropy of monoclinic Gd_2O_3. *The Journal of Chemical Thermodynamics* **37**, 1219–1225 (2005). https://doi.org:10.1016/j.jct.2005.02.014

18 Westrum, E. F. & Justice, B. H. Thermophysical properties of the lanthanide oxides. III. Heat capacities, thermodynamic properties, and some energy levels of Dysprosium(III), Holmium(III) and Erbium(III) oxides. *The Journal of Physical Chemistry* **67**, 659–665 (1963). https://doi.org:10.1021/j100797a029

19 Justice, B. H., Westrum, E. F., Chang, E. & Radebaugh, R. Thermophysical properties of the lanthanide oxides. IV. Heat capacities and thermodynamic properties of thulium(III) and lutetium(III) oxides. Electronic energy levels of several lanthanide(III) ions. *The Journal of Physical Chemistry* **73**, 333–340 (1969). https://doi.org:10.1021/j100722a010

20 Alvero, R., Bernal, A., Carrizosa, I., Odriozola, J. A. & Trillo, J. M. Lanthanide oxides: Thermochemical approach to hydration. *Journal of Materials Science* **22**, 1517–1520 (1987). https://doi.org:10.1007/bf01233156

21 Counsell, J. F., Dell, R. M. & Martin, J. F. Thermodynamic properties of uranium compounds. Part 2.—Low-temperature heat capacity and entropy of three uranium nitrides. *Trans. Faraday Soc.* **62**, 1736–1747 (1966). https://doi.org:10.1039/tf9666201736

22 Robie, R. A. & Hemingway, B. S. *Thermodynamic properties of minerals and related substances at 298.15 K and 1 bar (10^5 pascals) pressure and at higher temperatures* (1995).

23 Cox, J. D., Wagman, D. D., & Medvedev, V. A. *CODATA Key Values for Thermodynamics*. (1989).
